# Temperature-driven mechanistic transition in propylene oxidation over Pt/CeO_2_ ensemble catalysts

**DOI:** 10.1038/s41467-025-64243-y

**Published:** 2025-10-16

**Authors:** Zihao Li, Xingyan Chen, Yao Lv, Sheng Dai, Huazhen Chang, Zhenguo Li, Kailong Ye, Fudong Liu, Lei Ma, Naiqiang Yan

**Affiliations:** 1 State Key Laboratory of Green Papermaking and Resource Recycling, School of Environmental Science and Engineering, Shanghai Jiao Tong University, Shanghai, China; 2 PetroChina Petrochemical Research Institute, CNPC Company, Beijing, China; 3 Key Laboratory for Advanced Materials and Feringa Nobel Prize Scientist Joint Research Center, School of Chemistry & Molecular Engineering, East China University of Science and Technology, Shanghai, China; 4 School of Chemistry and Life Resources, Renmin University of China, Beijing, China; 5 National Engineering Laboratory for Mobile Source Emission Control Technology, China Automotive Technology & Research Center Co. Ltd., Tianjin, China; 6 Department of Chemical and Environmental Engineering, Bourns College of Engineering, Center for Environmental Research and Technology (CE-CERT), Materials Science and Engineering (MSE) Program, UCR Center for Catalysis, University of California, Riverside, CA USA

**Keywords:** Pollution remediation, Heterogeneous catalysis, Catalytic mechanisms

## Abstract

Pt/CeO_2_ ensemble catalysts are promising for propylene (C_3_H_6_) oxidation in vehicle exhaust, yet identifying the intrinsic active sites and understanding how the metal-support interface evolves at varying reaction temperatures remains contentious. Herein, we demonstrate that H_2_-activated Pt/CeO_2_ ensemble catalysts feature metallic Pt ensembles as intrinsic active sites, lowering the 50% conversion temperature by 120 °C after hydrogen activation. Various operando characterization techniques reveal an approximately 170 °C threshold temperature for the dynamic change of the reaction models. Meanwhile, kinetics and theoretical analysis illustrates that oxygen-facilitated dehydrogenation of *sp*^3^ C-H bonds is the rate-determining step. At low temperatures, both C_3_H_6_ and O_2_ adsorb and activate on metallic Pt, without CeO_2_ involvement. Once the temperature exceeds threshold, C_3_H_6_ fully covers Pt sites, while O_2_ activates over Pt-O-Ce interfaces and participates in dehydrogenation. This study highlights the dynamic nature of oxygen activation, leading to distinct reaction temperature regimes during C_3_H_6_ oxidation.

## Abstract (web summary)

Pt/CeO_2_ ensemble catalysts show great potential for hydrocarbon oxidation, but the nature of active sites and interfacial evolution during reaction has been debated. Here, the authors demonstrate metallic Pt ensembles serve as the true active sites for C_3_H_6_ oxidation, and confirm dynamic nature of oxygen activation with the threshold temperature around 170 °C.

## Introduction

Pt is recognized as one of the most active components for diesel oxidation catalysts and three-way catalysts in vehicle emission control^1,2^. CeO_2_ has been commonly used as the supporting materials for Pt-based emission control catalysts, due to the decent oxygen storage capacity and excellent reducibility^1^. Wherein, the defect sites and sufficient active oxygen species at the interfacial sites contributed to anchoring Pt active species and promoting low-temperature oxidation activity^3,4^. In the past decade, atomically dispersed Pt catalysts received much attention owing to the maximum utilization of precious metals. Especially, Pt single-atom catalysts (Pt SACs) exhibited outstanding reactivity towards CO oxidation^5,6^. However, the absence of Pt ensemble sites (Pt_e_) still constrains the catalytic activity towards alkene (such as C_3_H_6_) oxidation^7^. Therefore, great efforts have been devoted to fabricating Pt ensemble catalysts that could effectively accomplish the cleavage of C–H methyl or C = C double bonds during C_3_H_6_ oxidation^8,9^. It is still challenging to elucidate the intrinsic active species of Pt_e_/CeO_2_ ensemble catalysts and clarify the specific reaction pathways for the catalytic oxidation of C_3_H_6_.

The diversity in valence states (electronic properties) was one of the decisive aspects determining the reactivity of Pt_e_ catalysts, besides considering the geometry and size as critical factors. So far, it remains debated the oxidation state in charge of the intrinsic activity of atomically dispersed Pt catalysts. It was reported that Pt^0^ or Ptᵟ^+^ were the intrinsic active sites for catalytic oxidation of C_3_H_6_ from the previous literature^10–12^, which might be strongly affected by the catalyst support and promoters. The contentious issue of the oxidation state of Pt catalysts has also been investigated with CO oxidation, with intrinsic similarity to C_3_H_6_ oxidation. For example, Maurer et al. indicated that ${\mathrm{Pt}}_{{\rm{X}}}^{{\rm{\delta }}+}$ forming during catalytic oxidation of CO were the exclusive active sites^13^. Ding et al., however, discovered that Pt^0^ was the sole active phase and Ptᵟ^+^ was the spectator for catalytic oxidation of CO^14^. Therefore, complementary investigation of intrinsic Pt_e_ species with specific electronic properties could provide a guide for the rational design of highly efficient Pt_e_ catalysts.

Regarding the reaction pathway of catalytic oxidation of C_3_H_6_, either the reactive oxygen species (superoxide and peroxide) transformed from gaseous O_2_ or lattice oxygen from interfacial sites might engage in catalytic oxidation of C_3_H_6_. On one hand, C_3_H_6_ could adsorb over the catalyst surface, and the reaction took place between the C_3_H_6_ molecules and reactive oxygen species to form intermediates^10,15^. On the other hand, C_3_H_6_ oxidation might follow the Mars-van Krevelen mechanism, in which C_3_H_6_ oxidation could react with lattice oxygen, causing the appearance of anion vacancies, followed by the re-oxidation of catalysts by gaseous oxygen in a separate step^16^. Yet, it might be subjective to directly conclude the catalytic oxidation reaction model without considering the reaction temperatures. For instance, the dynamic toluene oxidation mechanism variation was triggered by the activation of the different active oxygen species^17,18^. In the low-temperature regime, gaseous oxygen molecules were directly converted to adsorbed oxygen species to facilitate the toluene oxidation^17,18^. With the increased temperature, the lattice oxygen from the bulk phase of CeO_2_ supports gradually migrated to the interface and acted as the active surface lattice oxygen species to drive toluene oxidation^17,18^. A similar trend was detected for a dynamic transition of reaction mechanism from Langmuir-Hinshelwood to Mars-van Krevelen for toluene oxidation with rising temperature^19^. Furthermore, Li et al. found that the oxygen vacancy at the interfacial sites between Pt ensembles and CeO_2_ was inactive in the low-temperature domain for the water-gas shift reaction. Once the temperature exceeded 180 °C, oxygen vacancy-Ce^3+^ sites were stimulated and imitated to convert and supply active oxygen species to the interface^20^. Based on the above research, there might be a threshold temperature determining the oxygen activation to participate in the C_3_H_6_ oxidation reactions over Pt_e_/CeO_2_ catalysts. It is valuable to investigate the dynamic evolution properties to explicit the possible change in the reaction mechanism during the different temperature ranges. The results will benefit the understanding of the specific catalytic roles of Pt_e_ catalysts in emission control applications.

Herein, Pt ensembles were loaded over the CeO_2_ supports (Pt_e_/CeO_2_) via incipient wetness impregnation, which was further activated by H_2_ reduction to improve the catalytic performance for C_3_H_6_ oxidation. High-angle annular darkfield scanning transmission electron microscopy (HAADF-STEM), extended X-ray adsorption fine structure (EXAFS), X-ray photoelectron spectroscopy (XPS), and catalytic performance tests unraveled that H_2_ activation constructed metallic Pt ensembles locating at upper tiers of CeO_2_ serving as the intrinsic active sites. In situ Raman spectra, near ambient pressure X-ray photoelectron spectroscopy (NAP-XPS), and Electron energy loss spectroscopy (EELS) results demonstrated that gaseous oxygen was activated at Pt-O-Ce interfacial sites to promote C_3_H_6_ oxidation above 170 °C, acting as the threshold temperature. In situ Diffuse Reflectance Infrared Fourier Transform Spectroscopy (DRIFTS), rigorous kinetic studies, and Density Functional Theory (DFT) calculations affirmed that the C_3_H_6_ coverage change and oxygen activation at the interfacial sites caused the dynamic transformation of the reaction models. The results will guide the precise design of Pt_e_ catalysts and be also helpful for the understanding of their catalytic roles under varying reaction temperatures.

## Results

### Evaluation of the catalytic performance of Pt_e_/CeO_2_ catalysts

**Fig. 1:**
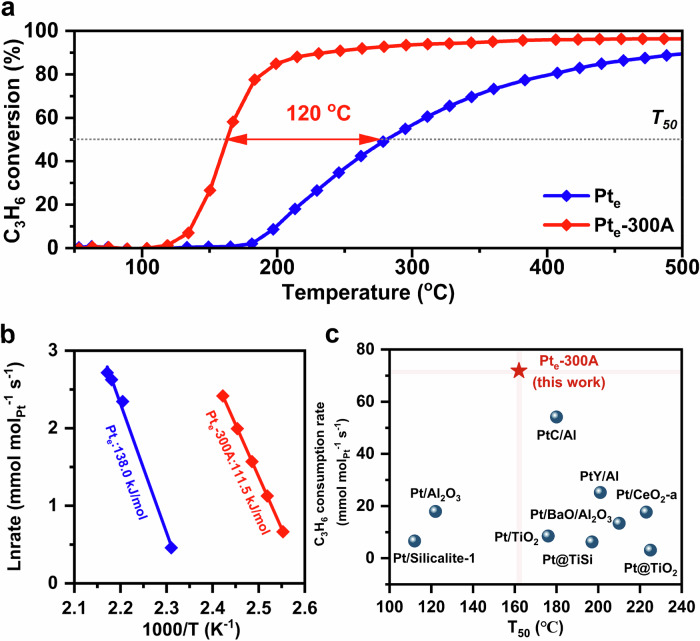
Catalytic activities and apparent activation energies of C_3_H_6_ oxidation over supported Pt catalysts. **a** C_3_H_6_ oxidation light-off curves. Reaction condition: 1000 ppm C_3_H_6_, and 10% O_2_ in N_2_ balance with a WHSV of 240,000 mL g^−1^ h^−1^; **b** Arrhenius plots of C_3_H_6_ oxidation; **c** Comparison of the reactivity of C_3_H_6_ oxidation between Pt_e_-300A and previously reported Pt-based catalysts. For details for comparison, see Supplementary Table 1.

The study first tried to measure C_3_H_6_ oxidation light-off performance over H_2_-activated Pt_e_/CeO_2_ catalysts, which significantly boosted the catalytic oxidation performance and could help explore the transformation of Pt ensemble structure and size during H_2_ activation at different temperatures (Supplementary Fig. 1). As shown in Fig. 1a, Pt_e_ barely reached 50% conversion of C_3_H_6_ (*T*_50_) at approximately 282 °C, Meanwhile, Pt_e_-300A (Pt_e_ after H_2_ reduction pretreatment at 300 °C) could significantly shift T_50_ to low temperatures around 160 °C. According to Fig. 1b, H_2_ activation caused a decline of the apparent activation energies from 138.0 to 111.5 kJ/mol for Pt_e_ and Pt_e_-300A, respectively. It was noteworthy that Pt_e_-300A catalysts achieved an exceptional catalytic consumption rate for C_3_H_6_ oxidation compared to other Pt-based catalysts as shown in Supplementary Table 1, proving the considerable activity of activated Pt_e_-300A catalysts (Fig. 1c). A similar promoting trend of H_2_ activation was mirrored for the catalytic oxidation activities of C_3_H_6_ and/or CO, and the apparent activation energies of CO oxidation also dropped from 67.3 to 41.5 kJ/mol (Supplementary Figs. 2 and 3). Furthermore, the as-synthesized Pt_e_-300A catalysts showed the same order of magnitude in CO consumption rate as the previously reported Pt/CeO_2_ catalysts from Supplementary Table 2. The above results indicated that the H_2_ activation could successfully fabricate efficient Pt_e_/CeO_2_ oxidation catalysts.

The synergistic interaction between Pt ensembles and CeO_2_ was confirmed by comparing the catalytic performance between Pt/CeO_2_ and Pt/γ-Al_2_O_3_ catalysts. To explore the interface effect of Pt metals and CeO_2_ support, the catalytic performance of Pt/γ-Al_2_O_3_ with inert supports was also examined. Supplementary Fig. 4 exhibited that H_2_-activated Pt_e_-300A had better catalytic reactivity at a low-temperature regime than Pt_e_/γ-Al_2_O_3_-300A catalysts. It indicated that the synergistic interaction between Pt and reducible CeO_2_ supports was important in governing catalytic oxidation activity over Pt_e_-300A catalysts. It is noteworthy that the physically mixed Pt_e_/γ-Al_2_O_3_&CeO_2_-300A catalysts obtained comparable activity toward C_3_H_6_ and CO oxidation to Pt_e_/γ-Al_2_O_3_-300A catalyst. It implied that the proximity mattered for Pt_e_/CeO_2_ catalysts, and the synergistic interactions only occurred at the Pt-O-Ce interfacial sites. Moreover, H_2_ activation had little effect on the catalytic oxidation activity of bare CeO_2_ supports for C_3_H_6_ oxidation (Supplementary Fig. 5). The results revealed the transformation of Pt active sites after H_2_ activation and synergistic interactions over Pt-O-Ce interfacial sites were two key factors in elevating oxidation performance of Pt/CeO_2_ catalysts.

Water vapor is a common component as a key element influencing the performance and longevity of Pt-based emission control catalysts^21,22^. The co-feeding of 5% H_2_O did not reduce the light-off performance of C_3_H_6_ oxidation over Pt_e_-300A (Supplementary Fig. 6). The catalytic activity remained stable in the presence of 5% H_2_O, highlighting the promise of Pt_e_-300A in practical applications for vehicle emission control (Supplementary Fig. 7). Meanwhile, Pt_e_-300A catalysts was pretty stable without any change of Pt valence states during the stability tests, based on the XPS spectra of Pt 4*f* core-level analysis (Supplementary Fig. 8). No deactivation was observed for Pt_e_-300A samples after cycling tests, also confirming the thermal stable properties of H_2_-activated catalysts (Supplementary Fig. 9).

**Fig. 2:**
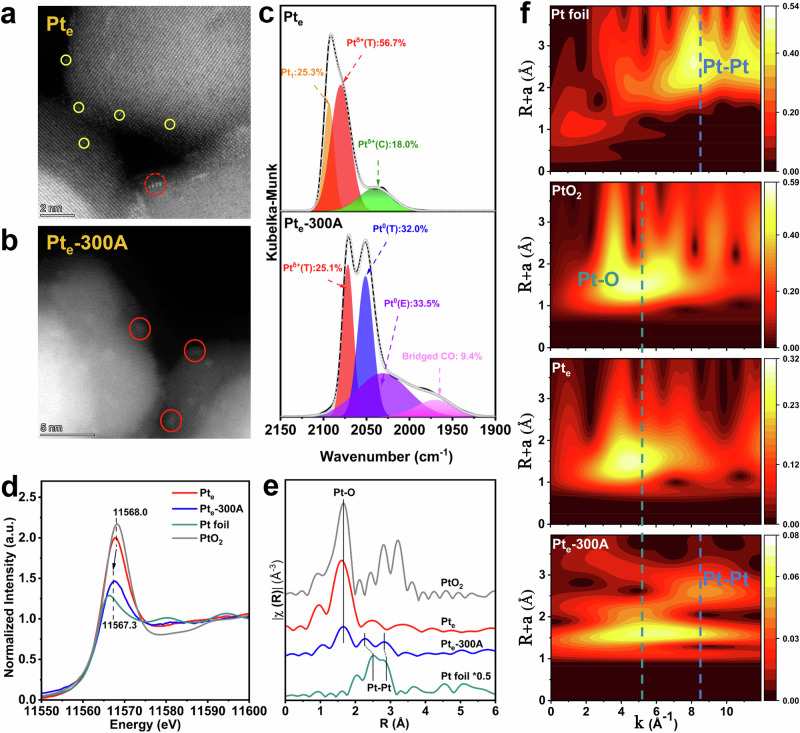
Structural characterization and identification of intrinsic active sites over Pt_e_ and Pt_e_-300A catalysts. HAADF-STEM images of **a** Pt_e_ and **b** Pt_e_-300A (yellow cycle: Pt single atoms; red dashed cycle: planner single-layer Pt ensembles; red solid cycle: multilayer Pt ensembles); **c** in situ DRIFTS spectra of CO adsorption at 30 °C on Pt_e_ and Pt_e_-300A; **d** normalized Pt L_3_-edge XANES and **e** Fourier-transformed *k*^2^-weighted EXAFS spectra in R space for Pt_e_ and Pt_e_-300A; **f** wavelet transform plot of Pt L_3_-edge EXAFS spectra for Pt foil, PtO_2_, Pt_e_, and Pt_e_-300A samples.

HAADF-STEM was carried out to analyze the geometry of Pt species on pristine and activated catalysts, which might be undergoing a dramatic reconstruction after H_2_ activation. A few Pt single atoms and a single-layer Pt ensemble with a mean diameter of 0.45 nm could be detected on pristine Pt_e_ catalysts (Fig. 2a and Supplementary Fig. 10a, b). H_2_ activation caused a transformation of Pt species to form multilayer Pt ensembles, with a mean diameter of 0.84 nm (Fig. 2b and Supplementary Fig. 10c, d). In situ DRIFTS experiments of CO adsorption were further performed to investigate the chemical states of surface Pt species. As shown in Fig. 2c, three major peaks at 2094, 2079, and 2036 cm^−1^ were deconvoluted from the IR bands on Pt_e_ catalysts, which could be ascribed to linearly bound CO adsorbed on Pt single atoms, the well-coordinated terrace sites, and the under-coordinated corner sites on Ptᵟ^+^ ensembles, respectively^23–25^. For Pt_e_-300A catalysts with hydrogen activation, an intense band with two sub-peaks at approximately 2051 and 2033 cm^-1^ appeared, corresponding to the linear CO adsorption at the well-coordinated terrace sites and under-coordinated edge sites within Pt^0^ ensembles, respectively^26–28^. The extra peak at 1983 cm^−1^ could be attributed to the bridging bound CO adsorbed on Pt ensembles^29^, affirming an enlarged Pt ensembles after H_2_ activation^30^. Moreover, the band at 2072 cm^−1^ remained on Pt_e_-300A as the CO linearly bound on unreduced oxidized Pt species. These newly created bands illustrated that the Pt ensembles over Pt_e_-300A catalysts were composed of both Pt^0^ and Ptᵟ^+^ sites.

The XAS data were further collected to elucidate the changes in the oxidation states and specific local coordination environments of Pt species after H_2_ activation. Figure 2d compared the X-ray absorption near edge structure (XANES) spectra between Pt_e_ and Pt_e_-300A catalysts using Pt foil and PtO_2_ as the reference samples. The edge position within Pt L_3_-edge XANES data of Pt_e_ was close to that of PtO_2_, suggesting the single-layer Pt ensembles mainly existed in the highly oxidized states. Furthermore, the edge position shifted to lower energy after H_2_ activation, suggesting a more reduced Pt state over Pt_e_-300A versus as-prepared Pt_e_ samples. As displayed in Fig. 2e, the EXAFS spectra were plotted in R space to investigate the local structures. Pt_e_ catalysts only demonstrated the first Pt-O coordination shell at 2.00 Å, implying that Pt ensembles were predominantly bound with the surface oxygen on CeO_2_ (100) facets. The fitting model of the Pt_e_-300A catalysts included both metallic Pt–Pt and Pt–O bonds, which indicated the formation of Pt^0^ ensemble sites over the top layer of Ptᵟ^+^ planar (Supplementary Figs. 11 and 12). Meanwhile, the shorter Pt-Pt bond distance over Pt_e_−300A (2.74 Å), compared to the Pt foil (2.76 Å), could be ascribed to the increment of the local electron density between two adjacent metal atoms triggered by the rehybridization of the *spd* orbitals in metal clusters^31,32^. This phenomenon usually occurs in the small metal nanoclusters^33^, coinciding with the relatively small average diameter of the Pt ensembles over Pt_e_−300A catalysts. Additionally, Fig. 2f showed the wavelet transform analysis based on Pt L_3_-edge EXAFS oscillations, where the Pt_e_-300A plots comprised Pt–O and Pt–Pt bonds. In contrast, only Pt-O bonds could be identified over Pt_e_ catalysts, revealing that H_2_ activation created metallic Pt ensembles as the major species, accompanied by partially oxidized Pt. Additional XRD and N_2_ physisorption results proved that H_2_ activation did not significantly affect the textural properties of CeO_2_ supports (Supplementary Figs. 13, 14).

XPS experiments were performed to measure the chemical states of the supported Pt species. As shown in Supplementary Fig. 15 and Supplementary Table 3, only Pt^2+^ and Pt^4+^ were detected on Pt_e_ samples, suggesting that highly oxidized Pt species exclusively survived on CeO_2_. Metallic Pt was formed after H_2_ activation, the deconvoluted doublets shifted to 71.8, 72.7, 75.1, and 76.0 eV, corresponding to Pt^0^ or Pt^2+^ in Pt 4*f*_7/2_ spectra and Pt 4*f*_5/2_ spectra, respectively^34,35^. H_2_-TPR profiles (Supplementary Fig. 16) further demonstrated a significant declination of the relative area in the range of 100–250 °C on Pt_e_-300A catalysts, indicating the disappearance of oxidized Pt species such as PtO_x_^36^, which agreed with the lowered coordination number of Pt–O bond between Pt_e_ and Pt_e_-300A (4.2 ± 0.2 vs. 1.5 ± 0.1) from Supplementary Table 4.

Since two different Pt sites, including Pt^0^ and Ptᵟ^+^, have been detected on Pt ensembles, it is unavoidable to directly compare the reactivity on these sites regarding C_3_H_6_ oxidation quantitatively, which would further validate the rationality of metallic Pt ensembles as the unique intrinsic active sites. On one hand, DFT calculations were first conducted to precisely analyze the free energy changes of the oxygen-facilitated dehydrogenation of the *sp*^3^ hybrid C-H bonds, which was recognized as the rate-determining step (RDS) for catalytic oxidation of propylene in the following section. As shown in Supplementary Figs. 17 and 18, Pt^0^ sites on the top layers obtained a much lower energy barrier (1.53 eV) than both Pt^δ+^ at the bottom sites (2.41 eV) and single-layer Pt ensembles on pre-activated Pt_e_ catalysts (1.68 eV) for the dehydrogenation process, which led to the much better catalytic activity of C_3_H_6_ oxidation on metallic Pt sites formed during the H_2_-trigged structural evolution. Additionally, it was consistent that the abstraction of the C-H bonds on the methyl group over metallic Pt ensemble sites exhibited a similar magnitude of activation energy barrier, compared to the identical process that took place on Pd/Cu_55_ clusters (1.43 eV)^37^ and Pt_2_Sn/Pt(111) surface (1.63 eV) catalysts^38^. Supplementary Fig. 19 demonstrated density of state (DOS) results based on the *d*-orbital of Pt ensembles for different sites, where the *d*-band of Pt^0^ was centered at a higher energy (−1.98 eV) compared to Ptᵟ^+^ (−2.58 eV), indicating an increment of the adsorption reactivity accompanied with the facilitation of C_3_H_6_ adsorption on metallic Pt sites^39^. As illustrated in Supplementary Fig. 20, the Bader charges of the upper-tier Pt^0^ and bottom-layer Ptᵟ^+^ over CeO_2_ surface were calculated to be −0.13e and +0.30e, respectively. It revealed that metallic Pt ensemble sites could provide many more electrons for the C_3_H_6_ adsorbed molecules. Moreover, the difference in charge density of the oxygen-facilitated dehydrogenation step illustrated a more frequent electron transfer over Pt^0^ than Ptᵟ^+^ sites, where the correlated results were affirmed by the charge density difference of 1.06e and 0.89e for −CH_3_ activation with the assistance of oxygen over Pt^0^ and Ptᵟ^+^ sites, respectively. Furthermore, DOS calculations for C_3_H_6_ adsorbed at Pt^0^ and Ptᵟ^+^ sites revealed distinct electronic interactions shown in Supplementary Fig. 21. At the Pt^0^ site, a significant orbital hybridization between C and Pt occurred within the energy range from −5 to −10 eV. The broad overlap across multiple energy levels indicated strong electron cloud interactions. The C-Pt orbital hybridization was also present over the Ptᵟ^+^ site, yet the hybridized peaks exhibited markedly reduced intensity and narrower energy distribution. Meanwhile, strong hybridization at the Pt^0^ site shifted the *d*-band center to lower energies to −3.02 eV, in comparison to −2.07 eV at the Ptᵟ^+^ site. Collectively, these results demonstrated more stable C_3_H_6_ adsorption at top adsorption sites and stronger interfacial interactions, thereby facilitating subsequent C_3_H_6_ activation. On the other hand, the comparison of inherent catalytic oxidation activity between Pt^0^ and Ptᵟ^+^ sites was also elucidated by FTIR tests, where CO was used as the titration gas to measure the reactivity of different Pt sites. The transient reactions were conducted between the saturated adsorbed CO and the flowing O_2_ (Supplementary Fig. 22). Metallic Pt exhibited a rapid CO consumption rate over Pt_e_-300A catalysts, confirming its role served as the sole intrinsic active site for catalytic oxidation, demonstrating significantly better reactivity than Ptᵟ^+^. The universality of the metallic Pt ensembles was further examined on Pt_e_/γ-Al_2_O_3_ catalysts. Supplementary Figs. 23–25 additionally confirmed a positive correlation between the increased catalytic oxidation reactivity and the rising ratio of metallic Pt sites regarding Pt_e_/γ-Al_2_O_3_ catalysts with H_2_ activation, validating that metallic Pt functioned as the intrinsic active sites on Pt ensemble clusters supported by γ-Al_2_O_3_.

### Investigation of interfacial property by in situ characterization techniques

**Fig. 3:**
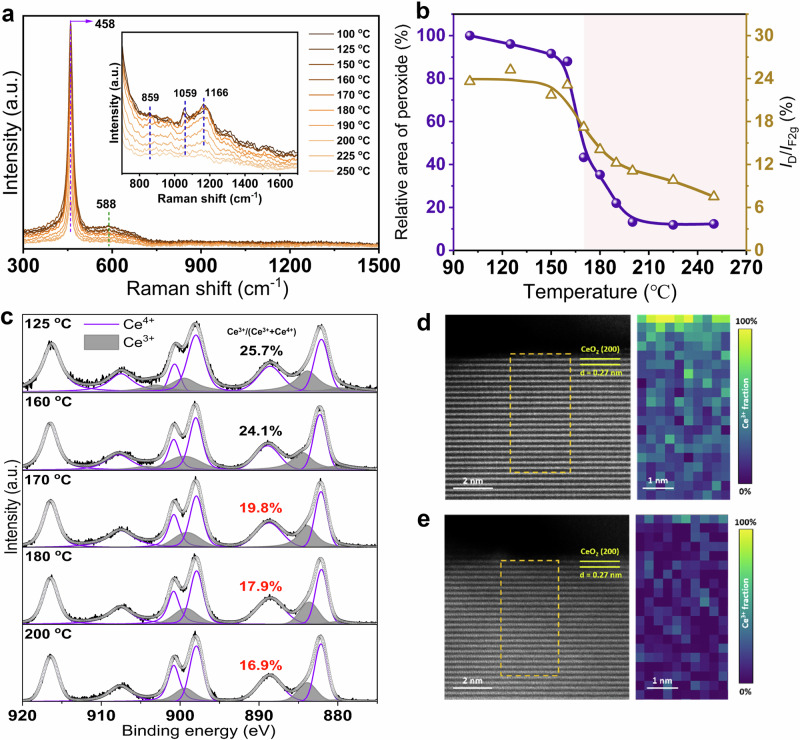
Dynamic change of Pt_e_-300A under C_3_H_6_ oxidation. **a** In situ Raman spectra of C_3_H_6_ oxidation over Pt_e_-300A from 100 to 250 °C; **b** Variation of peroxide ratio and I_D_/I_F2g_ ratio in the range of 100 to 225 °C; **c** NAP-XPS of Ce 3*d* spectra at different temperatures; **d**, **e** EELS result of Pt_e_-300A after catalytic reactions at 162 °C and 188 °C, respectively.

The dynamic change of the reaction model was studied using in situ characterization methods to uncover the mystery of C_3_H_6_ oxidation over Pt_e_-300A at different temperatures. Firstly, in situ Raman spectra were measured using the identical reaction conditions as light-off tests. Figure 3a, b demonstrated two prominent bands at 458 and 588 cm^−1^, symbolizing the F_2g_ symmetry mode of the CeO_2_ fluorite structure and the defect-induced mode, respectively^40,41^. Meanwhile, the band at 859 cm^−1^ could be assigned to peroxide species (O_2_^2−^)^40^, and the bands at 1059 and 1166 cm^−1^ could be assigned to the in-plane bend of C–H species^19^. The intensity ratio of *I*_*D*_/*I*_*F2g*_ remained constant around 23.0% as the temperature rose from 100 to 160 °C, revealing that the concentration of oxygen vacancies was relatively stable. Once the reaction temperature was further elevated and exceeded 170 °C, the oxygen vacancy defects were gradually annihilated due to the adsorption and activation of adsorbed oxygen molecules. At the same time, the concentrations of peroxide species were also decreased above 170 °C, which was in line with their catalytic roles in C_3_H_6_ oxidation. Moreover, an equivalent phenomenon was detected over CO oxidation in Supplementary Fig. 26, where the *I*_D_/*I*_F2g_ ratio abruptly dropped above 170 °C, confirming a similar oxygen activation process stimulated and participated in CO oxidation. Secondly, NAP-XPS spectra of Ce3*d* were displayed in Fig. 3c and Supplementary Fig. 27 to trace the transformation in chemical valence during the heating process. From 100 to 160 °C, the fraction of Ce^3+^ species was relatively stable at approximately 25%. When the reaction temperature increased above 170 °C, the Ce^3+^ concentration gradually declined. It illustrated that the ratio of Ce^3+^/(Ce^3+^+Ce^4+^) showed a decreased trend with ramping reaction temperatures, implying that the oxygen vacancy defects over Pt-O-Ce interfacial sites were gradually replenished during the reaction at high temperatures. Lastly, the Ce^3+^ ratio at the interfacial sites after catalytic reactions below and above the threshold temperature of approximately 170 °C was probed by EELS to unambiguously verify the change of surface vacancies. As shown in Fig. 3d, e, the interfacial section of Pt_e_-300A catalysts exhibited a more pronounced yellowish hue in the color bar after the C_3_H_6_ oxidation reaction at 162 °C compared to that at 188 °C, indicating a higher Ce^3+^ ratio at the lower temperature. This observation suggests that oxygen vacancy defects were filled by activated oxygen during C_3_H_6_ oxidation with ramping temperature and cause a greater proportion of Ce^4+^ formation. By combining in situ Raman spectra, NAP-XPS, and EELS results, it could be concluded that 170 °C was the threshold temperature for C_3_H_6_ oxidation on Pt_e_-300A catalyst by different reaction pathways.

### Evaluation of C_3_H_6_ oxidation mechanism by varying reaction temperatures

**Fig. 4:**
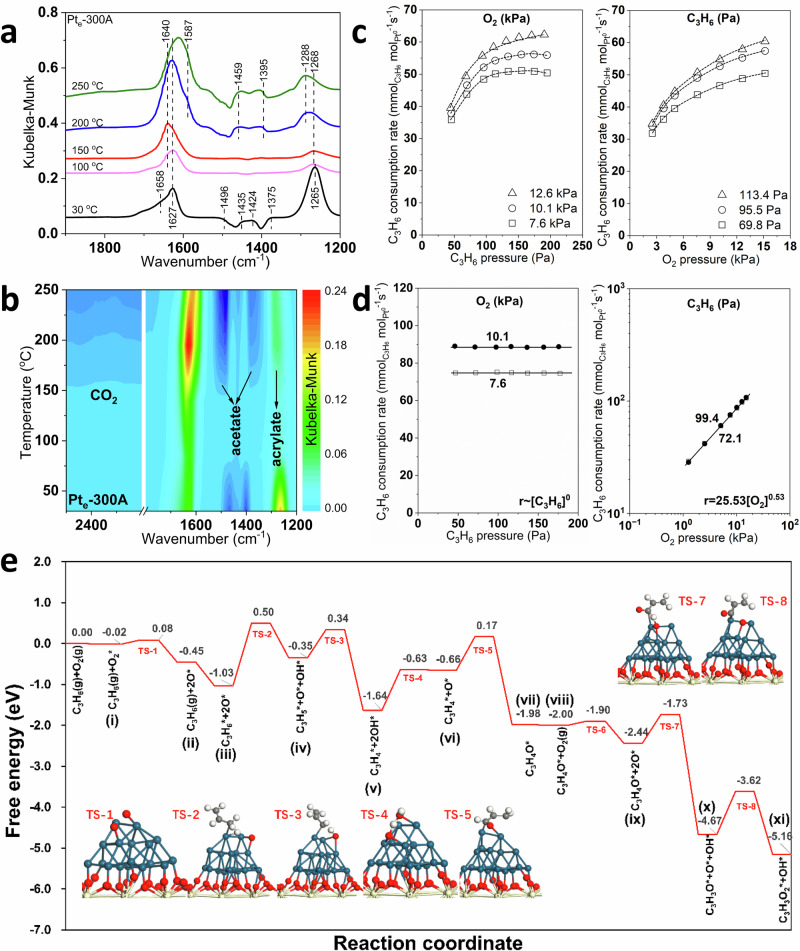
Study of the reaction mechanisms and surface intermediates for C_3_H_6_ oxidation over Pt_e_-300A. **a** In situ DRIFTS spectra of steady-state C_3_H_6_ and O_2_ co-adsorption; **b** Contour graphs for C_3_H_6_ oxidation; **c**, **d** Effects of C_3_H_6_ and O_2_ partial pressures on C_3_H_6_ consumption rate over Pt_e_-300A at 162 °C and 188 °C, respectively; **e** DFT calculations of C_3_H_6_ oxidation mechanisms and energy barriers with detailed transition states and free energy change.

In situ DRIFTS experiments of steady-state reactions at various temperatures were further conducted over Pt_e_ and Pt_e_-300A catalysts to elucidate different surface intermediates during C_3_H_6_ oxidation, and the detailed assignment of IR spectra was ascribed to Supplementary Table 5. As shown in Supplementary Fig. 28, the bands at 1660, 1274, and 1622 cm^−1^ were observed at 30 °C on Pt_e_, which could be attributed to C = C stretching, CH_2_ deformation of gaseous C_3_H_6_, and C = C stretching of the adsorbed C_3_H_6_ molecules, respectively^42–44^. Once the temperature was increased to 150 °C, gaseous C_3_H_6_ completely vanished, suggesting C_3_H_6_ activation at high temperatures. The characteristic bands at 1240 and 1284 cm^−1^ appeared when the reaction temperature reached 200 °C, corresponding to the generation of surface acrolein and acrylate^45,46^. These species would be further converted to acetate (1463 and 1405 cm^−1^)^46^. As shown in Fig. 4a, b, the IR spectra of Pt_e_-300A demonstrated complex adsorbed species at 30 °C. The bands at 1496 cm^−1^ and 1435 cm^−1^ were attributed to π-allylic intermediates, which were generated by the hydrogen abstraction from the weak methyl group with *sp*^3^ hybridization^47,48^. Once the temperature reached 100 °C, gaseous C_3_H_6_ still presented at *ca*. 1658 and 1265 cm^−1^, while acrolein at 1268 cm^−1^ was generated as the successive intermediates^46^. Acrylate exhibited intensive bands at approximately 1640 and 1288 cm^−1^, while acetate could be observed at 1459 and 1395 cm^−1^ above 200 °C^49^. The intensity of acetate was gradually increased with further ramping temperature, suggesting the facilitation of acetate generation at high temperatures. It should be noted that formate species might also be formed accompanying acetate generation, due to the destructive oxidation of C_3_H_6_ by breaking C = C bonds. Yet, no obvious formate species were detected in the IR spectra, probably due to the thermal decomposition above 200 °C. Moreover, compared to the situation on Pt_e_ sample, many distinctive bands of acetate were observed at corresponding temperatures, while the characteristic peaks of CO_2_ were only detected on the surface of Pt_e_-300A. Therefore, it suggested that the metallic Pt ensemble sites on H_2_-activated catalysts stimulated the oxidation process of acrylate to acetate, which could be recognized as a prior step in producing the final products of CO_2_ and H_2_O.

Rigorous kinetic studies were further conducted to evaluate the elementary reaction steps of C_3_H_6_ oxidation over Pt_e_-300A catalysts. Figure 4c exhibited a sub-linear dependence between the C_3_H_6_ consumption rate on the partial pressures of C_3_H_6_ and O_2_ at 162 °C, which revealed classic Langmuir-Hinshelwood models on Pt_e_-300A catalysts. Based on the kinetics data at 162 °C, the elementary steps of C_3_H_6_ oxidation could be deduced and summarized in Supplementary Fig. 29. Initially, the gaseous O_2_ was reversibly adsorbed on the vacancy sites (*) over metallic Pt ensemble sites (step 1), and then dissociated to produce the adsorbed O* atoms (step 2). Meanwhile, the quasi-equilibrium adsorption of C_3_H_6_ molecules occurred on the same sites and generated C_3_H_6_* (step 3). Subsequently, the dissociated O* kinetically activated the C-H bond by irreversibly coupling with the C_3_H_6_* to facilitate dehydrogenation and form surface-adsorbed C_3_H_5_* and OH* (step 4), which was recognized as RDS in the whole reaction process. The kinetics models of oxygen-facilitated dehydrogenation were similar to the catalytic oxidation of C_3_H_6_ over Ag/Al_2_O_3_ cluster catalysts^50^. The following process was induced by the quasi-equilibrated interaction between surface C_3_H_5_* and O*, generating adsorbed CO_2_* and OH* (step 5). Ultimately, the final product of CO_2_ was desorbed from the catalyst surface (step 6), and H_2_O was generated from the reaction between OH* molecules and then desorbed by leaving the vacancy sites (steps 7 and 8). The derivation of Supplementary Equation (1) was mainly based on the assumption of the pseudo-steady state for the kinetically observable O_2_*, O*, and C_3_H_6_* species, accompanied by the quasi-equilibrium for steps 1–3 previous to the RDS. Supplementary Table 6 summarized the kinetic parameters derived by regressing the kinetics data to Supplementary Equation (1) while minimizing the residuals. Generally, C_3_H_6_ oxidation pathways started from the adsorbed and activation of C_3_H_6_ and O_2_. Then, the reactions underwent C–H scission with O* to form an allyl intermediate (C_3_H_5_*), which was considered as kinetically-relevant step for the whole reaction.

DFT calculations were further conducted to explore the change of energies in the specific reaction route based on the elementary steps, and Supplementary Table 7 summarizes the potential energies in every reaction step. As shown in Fig. 4e, the reaction was initiated by the adsorption of O_2_ on metallic Pt sites and then dissociated to the adsorbed O* molecules with a low dissociation energy of 0.10 eV (TS-1). The adsorbed O* was subsequently reacted with C_3_H_6_* adsorbed on metallic Pt ensembles with the activation energy of 1.53 eV (TS-2). Subsequently, the dehydrogenated C_3_H_5_* was further activated by O* to generate C_3_H_4_* and OH* with an energy barrier of 0.69 eV (TS-3). After the formed OH* desorbed from the surface of metallic Pt ensembles with the energy of 1.01 eV (TS-4), the dissociated O* would interact with C_3_H_4_* to generate the surface-adsorbed acrolein (C_3_H_4_O*) with the reaction energy calculated as 0.83 eV (TS-5). Afterward, the second O_2_ dissociation process occurred, with an identical activation energy to TS-1, to generate more O* species (TS-6), which was then coupled with C_3_H_4_O* to form C_3_H_3_O* intermediates with the reaction energy of 0.71 eV (TS-7). Next, C_3_H_3_O* acted as the crucial precursor of the carboxylates and was further coupled with the dissociated O* to generate activated acrylate species (C_3_H_3_O_2_*) with an activation energy of 1.05 eV (TS-8). Lastly, CO_2_* and H_2_O* were produced and desorbed as the final products with a total free energy of −18.21 eV.

**Fig. 5:**
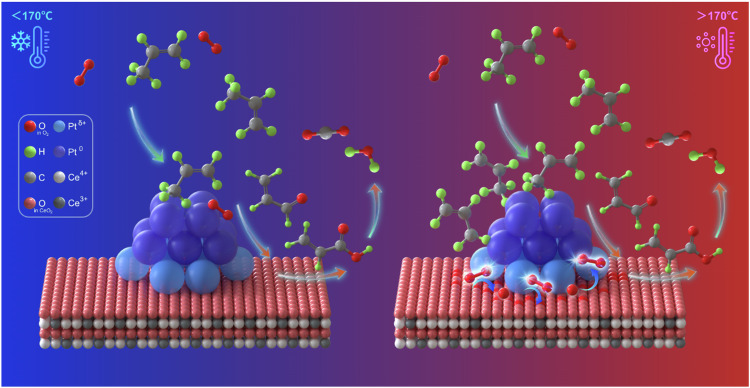
The schematics of dynamic reaction pathways of C_3_H_6_ oxidation over Pt/CeO_2_ ensemble catalysts. At low temperatures (<170 °C), the reactions followed the classic Langmuir-Hinshelwood model, where both C_3_H_6_ and O_2_ were adsorbed over metallic Pt ensembles. In contrast, at high temperatures (>170 °C), metallic Pt ensembles were fully covered by adsorbed C_3_H_6_, where O_2_ was adsorbed and activated at vacant sites of Pt-O-Ce interfaces without competition with C_3_H_6_.

It was demonstrated that the reaction pathways of C_3_H_6_ oxidation followed classic Langmuir-Hinshelwood models based on the above kinetics and DFT results. Notably, the kinetic model of C_3_H_6_ oxidation might be changed by elevating the reaction temperature to 188 °C. Figure 4d revealed a zero-reaction order for C_3_H_6_ partial pressure, while a dependence with the half-reaction order of 0.53 was observed for O_2_ partial pressure. The reaction order of approximately 0.5 regarding O_2_ partial pressure was also observed for the kinetic studies of CO oxidation over Pt_1_/CeO_2_^51^ and Rh/CeO_2_^52^ catalysts, which might prefer to occur on the supported catalysts with small metal clusters. It suggested that C_3_H_6_ molecules adsorbed stronger than O_2_ and fully covered the surface of metallic Pt ensemble sites at 188 °C. Meanwhile, the adsorption and dissociation of O_2_ took place at the vacant sites of the Pt-O-Ce interface (Pt-O_v_-Ce). Supplementary Fig. 30 listed the sequence of fundamental steps consistent with the observed kinetics data of C_3_H_6_ oxidation once the reaction elevated to 188 °C. Compared to Supplementary Fig. 29, the major difference was O_2_ adsorption and activation at the unoccupied oxygen vacancy (#) over Pt-O-Ce interfacial sites without competition with C_3_H_6_. Subsequently, the dissociated O^#^ at the interfacial sites reacted with the adsorbed C_3_H_6_* at the Pt ensembles to finish the dehydrogenation of the *sp*^3^ hybrid C-H bond and generate adsorbed C_3_H_5_* and OH^#^, which was irreversible and determined to be the RDS for the whole reaction. Supplementary Equation (2) could accurately describe the kinetics data of the reaction rates of C_3_H_6_ oxidation at 188 °C. Furthermore, the parity plots further verified the accuracy of the rate equation by comparing experimental and calculated data (Supplementary Fig. 31). The fractional coverages of *θ*(C_3_H_6_*) could be further calculated based on the obtained kinetics data and related partial pressures of the reactants. It was found that the fractional coverages were higher than 0.99 for *θ*(C_3_H_6_*) at temperatures of 188 °C, while the values would be only 0.20–0.55 for the same reaction at 162 °C. The results implied that the surface coverage of C_3_H_6_ over Pt^0^ active sites was much more significant with increasing reaction temperatures, while oxygen would be adsorbed and activated at the Pt-O-Ce interfacial sites without competition with surface C_3_H_6_. Based on the above results, it was reasonable to deduce that there should be a threshold temperature affecting the reaction model during C_3_H_6_ oxidation. According to the findings, the dynamic reaction models based on different reaction temperature ranges were summarized and illustrated in Fig. 5.

It should be noted that the C_3_H_6_ oxidation might not follow the Mars-van Krevelen model at 188 °C in this study. On one hand, the Mars-van Krevelen model typically initiated the reaction between C_3_H_6_ and activation of lattice oxygen, followed by the activated oxygen (O*) filling into the oxygen vacancies. The reaction order of approximately 0.5 regarding O_2_ partial pressure suggested the assumption that the elementary step of oxygen filling right after O* formation was the RDS. Yet, the fractions of Ce^3+^ would gradually decrease with ramping reaction temperatures and caused an increase of Ce^4+^ concentrations as evidenced by EELS tests and also in situ Raman and NAP-XPS study, suggesting that the oxidation of Ce^3+^ was accelerated in the high temperature ranges. It meant that the oxygen activation and filling into vacancies was a fast reaction at high temperatures, and would not govern the whole catalytic reaction rate. On the other hand, theoretical results simulated that oxygen was adsorbed and activated at the top layer and interfacial Pt-O-Ce sites, which exhibited the energy barriers of 0.10 and 0.22 eV, respectively (Supplementary Table 8). Meanwhile, the energy barrier of dehydrogenation facilitated by interfacial oxygen was observed as 1.53 and 1.68 eV for the corresponding sites, respectively. It suggested that the oxygen-facilitated dehydrogenation was still the RDS step for C_3_H_6_ oxidation.

## Discussion

A highly efficient Pt/CeO_2_ ensemble catalyst was obtained through a facile H_2_ activation, which exhibited potential application toward vehicle emission control. HAADF-STEM and EXAFS results suggested the reconstruction from Pt single-layer planar (0.45 nm) to multilayer ensemble (0.84 nm) with the top layers composed of Pt^0^ sites by hydrogen activation. In situ Raman spectra, NAP-XPS, and EELS experiments revealed that Ce^3+^ defects and dioxygen intermediates were activated once the reaction temperature exceeded 170 °C. Based on the complementary experiments and theoretical results, Pt^0^ ensemble sites demonstrated a low activation barrier for the dehydrogenation of *sp*^3^ hybrid C-H with the assistance of oxygen, identified as the RDS, compared to Ptᵟ^+^ sites. The kinetic results revealed that the C_3_H_6_ oxidation experienced a dynamic transformation of the reaction models, where the adsorption and dissociation of O_2_ occurred at interfacial Pt-O_v_-Ce sites after the temperature surpassed the threshold at approximately 170 °C. Combining the experimental phenomena, it was deduced that the involvement of interfacial Pt-O_v_-Ce sites was the key to triggering the dynamic evolution of the reaction models. Generally, this research not only successfully clarified that the metallic Pt ensembles were the intrinsic active sites for the highly efficient Pt/CeO_2_ catalysts regarding C_3_H_6_ oxidation, but also elucidated the dynamic change of the interfacial properties and the reaction model using in situ characterization methods. This work will guide the precise design and optimization of effective atomically dispersed platinum-based catalysts for emission control applications.

## Methods

### Catalyst preparation

#### Materials

All the chemicals are analytical reagent (AR) grades unless otherwise stated. Cerium nitrate hexahydrate (Ce(NO_3_)_3_·6H_2_O) and sodium hydroxide (NaOH) powder were purchased from Sinopharm Chemical Reagent Co., Ltd. The noble metal precursor of tetraammineplatinum (II) nitrate (Pt(NH_3_)_4_(NO_3_)_2_) with 99.995% trace metals basis was obtained from Sigma-Aldrich. The γ-Al_2_O_3_ was bought from Sasol Chemical LLC. The deionized water was directly produced and collected via a Milli-Q ® water purification machine from Merck KGaA.

### Preparation of CeO_2_ supports

A hydrothermal method was applied to synthesize supporting CeO_2_ nanocubes^53^. 16.88 g NaOH and 1.96 g Ce(NO_3_)_3_·6H_2_O were dissolved in 30 mL and 40 mL deionized water, respectively. After magnetic stirring for 15 min, the NaOH solution was added dropwise to Ce(NO_3_)_3_·6H_2_O solution under vigorous stirring for another 30 min. Subsequently, the mixed solution was transferred to a 100 mL Teflon bottle, which was then tightly sealed in a stainless-steel vessel autoclave and hydrothermally treated at 180 °C for 24 h. After cooling to room temperature, the white precipitate was washed and collected by centrifuging with deionized water at least three times and vacuum dried at 80 °C for 12 h. Finally, the yellowish products were calcined at 500 °C for 4 h with a ramping rate of 1 °C min^−1^ to obtain CeO_2_.

### Preparation of Pt_e_/CeO_2_ ensemble catalysts

0.5 wt.% Pt species were loaded on CeO_2_ nanocubes via an incipient wetness impregnation method. 71 μL Pt(NH_3_)_4_(NO_3_)_2_ solution ([Pt] = 25 g L^−1^) was applied as the Pt precursor and diluted in 0.9 mL deionized water. The aqueous solution was sonicated and slowly dripped onto 0.25 g ground CeO_2_ powder. Subsequently, the mixture was evaporated at 60 °C for 1 h and oven-dried at 110 °C for 8 h. The resulting powder was calcined in static air at 500 °C for 6 h with a temperature ramp of 1 °C min^−1^ to produce Pt ensemble nanoclusters denoting as Pt_e_. For the hydrogen activation process, Pt_e_ was pretreated in 10% H_2_/N_2_ reducing flow under different temperatures for 1 h. The pretreated samples were denoted as Pt_e_-XA, where X represented the activation temperature. Pt_e_ catalysts were pretreated at 100, 200, 300, or 400 °C to identify the optimal activation condition. As evidenced in Supplementary Fig. 1, Pt_e_-300A exhibited the highest catalytic activity towards C_3_H_6_ oxidation among the series of Pt_e_-XA catalysts.

### Preparation of Pt_e_/γ-Al_2_O_3_ ensemble catalysts

To conduct a comparison study, 0.5 wt% Pt_e_/γ-Al_2_O_3_ catalysts were prepared by the identical procedure to Pt_e_ catalysts. Prior to the synthesis, the commercial γ-Al_2_O_3_ was pretreated in a reducing flow of 10% H_2_ with N_2_ as the carrier gas at 350 °C for 2 h, leading to the formation of unsaturated Al^3+^_penta_ sites^54,55^.

### Catalytic activity evaluation

#### Light-off tests

Reactant gases including 1% C_3_H_6_/N_2_ (99.999%), 5% CO/N_2_ (99.999%) high-purified O_2_ (99.999%), and high-purified N_2_ (99.999%) were purchased from Shanghai Weichuang Standard Gas Analytical Technology Co., Ltd. The gas flow was precisely manipulated via mass flow controllers from Beijing Sevenstar Electronics Co., Ltd.

The catalytic light-off performance of C_3_H_6_ oxidation was measured in a fixed-bed quartz tube reactor with an internal diameter of 5.0 mm. Two thermocouples located upstream and downstream of the catalyst bed were utilized to monitor and regulate the temperature in the reactor. During each light-off test, the furnace reached the target temperature with a ramp rate of 2 °C min^−1^. 50 mg granulated catalysts with mesh sizes of 180–250 μm were diluted by the same size quartz sands, which was pre-calcined at 800 °C for 6 h in static air before mixing, to minimize the heat effect. The total flow rate was maintained at 200 mL min^−1^ for all reactions under atmospheric pressure, which corresponded to a weight hourly space velocity (WHSV) of 240,000 mL g^−1^ h^−1^. For C_3_H_6_ oxidation activity test, a feeding gas flow composed of 0.1% C_3_H_6_, 0.4% CO (when used), 5% H_2_O (when used), 10% O_2_ balanced with N_2_ passed through the tube reactor. The reactant and product gas concentrations were collected and analyzed by a Fourier-transform infrared (IR) spectrometer (Antaris IGS Gas Analyzer, Thermo Fisher Scientific Inc.). A high-pressure syringe pump was applied to precisely manipulate the injection rate of deionized water, which was completely vaporized in a gasifier isothermal at 150 °C before pumping into the reactor system. The C_3_H_6_ and CO conversions ($X$) were calculated by the following equations:

1 $${X}_{{{\rm{C}}}_{3}{{\rm{H}}}_{6}}(\%)=\frac{{C}_{{{\rm{C}}}_{3}{{\rm{H}}}_{6},\mathrm{in}}-{C}_{{{\rm{C}}}_{3}{{\rm{H}}}_{6},\mathrm{out}}}{{C}_{{{\rm{C}}}_{3}{{\rm{H}}}_{6},\mathrm{in}}}\times 100\%$$

2 $${X}_{\mathrm{CO}}(\%)=\frac{{C}_{\mathrm{CO},\mathrm{in}}-{C}_{\mathrm{CO},\mathrm{out}}}{{C}_{\mathrm{CO},\mathrm{in}}}\times 100\%$$

where C_in_ and C_out_ represent the inlet and outlet concentrations of the reactants, respectively.

C_3_H_6_ and CO consumption rates (mmol mol_Pt_^−1^ s^−1^) were collected at the constant temperature points and calculated by the following equation:

3 $$R=\frac{v\times {M}_{\mathrm{Pt}}}{{m}_{\mathrm{Pt}}\times {V}_{{\rm{m}}}}\times X$$

Where $v$ represents the flow rate of the reactants (mL s^−1^); ${M}_{\mathrm{Pt}}$ is the atomic mass of platinum; ${m}_{\mathrm{Pt}}$ is the mass of the platinum in the catalysts and determined by ICP-OES methods; ${V}_{{\rm{m}}}$ is the molar volume of gas equals 24.5 L mol^−1^ at room temperature.

### Apparent activation energy and kinetic evaluation

Arrhenius plots were constructed in a differential reactor by testing the C_3_H_6_ consumption rate at various temperature points following the identical reaction gas composition and WHSV to the light-off tests. All the catalytic conversions were strictly restricted below 15% to exclude the heat and mass transfer limitations. Meanwhile, the reactor was ramped to each target temperature at 2 °C min^−1^ and held for at least 45 min to achieve a steady state. The apparent activation energy (*E*_a_) was calculated by the Arrhenius plot.

For the kinetic experiments, the C_3_H_6_ oxidation rate was measured at a constant temperature by altering the partial pressure of the reactant gas without any back pressure in the reactor system. Heat and mass transfer limitations have been ruled out based on the theoretical results (Supplementary Table 9). 20 mg fine-ground catalysts with mesh sizes of 180–250 μm were mixed with 50 mg identical-size quartz sands, and packed into the fixed-bed tube reactor. Most catalytic conversions were restrained below 12%, maximizing at approximately 18%, which ensured that the kinetic evaluation could presume the differential reaction conditions. The mean partial pressure was determined as the average pressure between the inlet and outlet flows using the following equations:

4 $${P}_{{{\rm{C}}}_{3}{{\rm{H}}}_{6}}=\frac{{P}_{{{\rm{C}}}_{3}{{\rm{H}}}_{6},\mathrm{in}}+{P}_{{{\rm{C}}}_{3}{{\rm{H}}}_{6},\mathrm{out}}}{2}$$

5 $${P}_{{{\rm{O}}}_{2}}=\frac{{P}_{{{\rm{O}}}_{2},\mathrm{in}}+{P}_{{{\rm{O}}}_{2},\mathrm{out}}}{2}$$

The mean partial pressure of C_3_H_6_ and O_2_ ranged from 45.52 to 195.04 Pa and 2.51 to 12.64 kPa, respectively.

### Inductively coupled plasma optical emission spectroscopy (ICP-OES)

The ICP-OES experiments were conducted on Avio 550 (PerkinElmer Inc.) to detect the actual Pt content of different catalysts.

### Scanning transmission electron microscopy (STEM)

The aberration-corrected scanning transmission electron microscopy was conducted on a Thermo Fisher Themis Z transmission electron microscope to analyze the morphology and elemental distribution of the catalysts. This instrument was operated at a working voltage of 300 kV and equipped with two aberration correctors. 4 in-column Super-X detectors were applied to conduct Energy-dispersive X-ray spectroscopy analysis. High angle annular dark-field (HAADF) STEM images were captured using a camera length of 115 mm on HAADF detectors with inner and outer collection angles of 47 and 200 mrad, respectively. EELS data were acquired using a Gatan Enfinium ER (model 977) EELS spectrometer with a dual EELS function.

### X-ray absorption spectroscopy (XAS)

The XAS scans were performed for Pt_e_ and Pt_e_-300A on 21 A X-ray nanodiffraction beamline (4-bounce channel-cut Si (111) monochromator) of Taiwan Photon Source (TPS) at the National Synchrotron Radiation Research Center (NSRRC). The measurements for the Pt-L_3_ edge were carried out in the fluorescence mode in an energy range from 6 to 27 eV corresponding to the photon flux between 1 × 10^11^ ~ 3 × 10^9^ photon/s. The end-station is equipped with three ionization chambers and a Lytle/SDD detector after the focusing position of the KB mirror to collect data. The XANES data and EXAFS data were analyzed and fitted using the Athena and Artemis software from the Demeter software package, respectively.

### X-ray diffraction (XRD)

The XRD experiments were carried out on a LabX XRD-6100 instrument from Shimadzu Corporation, which operated at 40 mA and 40 kV with Cu Kα radiation (*λ* = 0.15406 nm) under an ambient condition. The scanning range of 2*θ* angle was recorded from 5 to 90° in a step rate of 3.33° min^−1^ to investigate the phase structure.

### N_2_ adsorption and desorption isotherm

N_2_ physisorption isotherm experiments were carried out on a Micromeritics ASAP 2020 analyzer. Prior to each measurement, the samples were firstly degassed under 300 °C for 8 h. The Barrett–Joyner–Halenda method was applied to determine the pore size distribution using the data of nitrogen desorption isotherm. The specific surface area was calculated using the Brunauer–Emmett–Teller equation.

### X-ray photoelectron spectroscopy (XPS)

The XPS spectra were collected on a Kratos AXIS Ultra DLD instrument (Shimadzu Corporation) operating at a working current and voltage of 8 mA and 14 kV, respectively, utilizing a monochromatic Al source. The binding energies of all tested elements were calibrated on the base of the standard C1*s* line at 284.8 eV.

### H_2_ temperature-programmed reduction (H_2_-TPR)

H_2_-TPR experiments were performed on the AutoChem II 2920 chemisorption analyzer from Micromeritics to investigate the redox properties of the catalysts. 40 mg catalysts were loaded into the U-shape quartz tube, which was pretreated in a 50 mL min^−1^ Ar flow at 300 °C for 30 min and then cooled down to room temperature. Once the baseline was stable at room temperature, the reactor was heated to 1000 °C at a ramp rate of 10 °C min^−1^ under a reducing gas stream of 5% H_2_/N_2_ flow (50 mL min^−1^). The signal was monitored and recorded with a thermal conductivity detector. For Pt_e_-300A samples, an additional activation process was conducted after the Ar purge at 300 °C, and the catalyst was pretreated in a 5% H_2_/Ar at the target temperature for 1 h prior to cooling to room temperature.

### In situ diffuse reflectance infrared Fourier transform spectroscopy (in situ DRIFTS)

In situ DRIFTS experiments were conducted on a Nicolet 6700 FTIR equipped with a mercury-cadmium-telluride detector cooling by liquid nitrogen. The fine-ground catalyst powder was loaded into a high-temperature reaction chamber with three Ba_2_F windows on the dome of the cell. The IR spectra resulted from averaging 64 scans at a resolution of 4 cm^−1^.

Pt_e_ and Pt_e_-300A samples were pretreated in the N_2_ flow (100 mL min^−1^) and 10% H_2_/N_2_ (100 mL min^−1^) at 300 °C for 1 h, respectively. Subsequently, the reaction chamber was cooled down to the target temperatures under N_2_ gas flow. The background spectrum was recorded at each desired temperature and subtracted from the sample spectrum.

For the CO adsorption and O_2_ purging experiments, 1% CO/N_2_ in a total flowrate of 100 mL min^−1^ was passed through the reaction chamber at the desired temperature. Once the CO adsorption saturated on the surface of catalysts, the system was purged by 100 mL min^−1^ N_2_ flow until the spectra became unchanged to remove the weakly adsorbed CO molecules. Finally, 10% O_2_ was introduced into the reactor to investigate the reactivity of different active sites. DRIFTS spectra were recorded throughout the entire reaction process. For the C_3_H_6_ oxidation, a mixture gas flow composed of 0.4% C_3_H_6_ and 10% O_2_ in N_2_ balance (100 mL min^−1^) was introduced into the reaction cell. The DRIFTS spectra were recorded at each temperature for at least 30 min.

### In situ Raman spectroscopy

A Horiba LabRam HR spectrometer using visible laser excitation with a wavelength of 514 nm emitted by a He-Cd laser was utilized for the in situ Raman experiments. A confocal microscope (Olympus BX-30-LWD) paired with a 50x long working distance objective was applied to focus the laser on the sample. The scattered photons were concentrated onto a single-stage monochromator and monitored using a UV-sensitive liquid nitrogen-cooled charge-coupled device (CCD) detector (Horiba CCD-3000 V). Pt_e_-300A samples were pretreated in 10% H_2_ at the tube reactor before the test. For C_3_H_6_ oxidation, the experiments were performed under the flowing reactant composed of 1000 ppm C_3_H_6_ and 10% O_2_ balanced with N_2_ at a total flowrate of 200 mL min^−1^. For CO oxidation, the experiments were conducted under the flowing reactant composed of 4000 ppm CO and 10% O_2_ balanced with N_2_ at a total flowrate of 50 mL min^−1^. Every spectrum was collected after the reaction for 20 min at each temperature point from 50 to 250 °C.

### NAP-XPS

The NAP-XPS were acquired using a SPECS-AU190069 instrument. The instrument features a multi-stage differential pumping system and a static voltage lens, making it suitable for usage in ultra-high vacuum (1 × 10^−9^ mbar) with gases ranging from 0 to 5 mbar. The spectra were obtained using monochromatized Al Kα irradiation (1486.6 eV), generated by 50 W of excitation source power in an Al anode (SPECS XR-50). The X-ray spot was approximately 0.3 mm in diameter and located near the nozzle’s opening. A pressure-reducing valve maintained a reaction pressure of 1 mbar. The powder sample was flattened into a smooth sheet and placed on a specially designed sample table that may be heated during the reaction. An electron flood cannon was used to correct for the charging of catalysts during tests.

### Computational method

The DFT calculations were performed by the Vienna Ab initio Simulation Package (VASP 5.4.1)^56^ with the projector augmented wave method^57^. The exchange-functional is treated using the generalized gradient approximation with Perdew-Burke-Emzerhof ^58^ functional. The energy cutoff for the plane wave basis expansion was set to 450 eV. Partial occupancies of the Kohn−Sham orbitals were allowed using the Gaussian smearing method and a width of 0.2 eV. The Brillouin zone was sampled with the Monkhorst–Pack k-point of 2 × 2 × 1 was applied for all the calculations for surface structures. The CeO_2_(100) support was modeled as a four-layer slab, with the top two layers fully relaxed and the bottom two layers constrained. The pre-activated surface was represented by a supported single-layer Pt_7_ cluster, while the post-activated state was modeled by a multi-layer Pt_17_ cluster. The self-consistent calculations apply a convergence energy threshold of 10^−5^ eV, and the force convergency was set to 0.05 eV/Å. The free energy corrections were calculated by the following equation:

6 $$\Delta {\rm{G}}=\Delta {\rm{E}}+{\Delta {\rm{G}}}_{{\rm{ZPE}}}+{\Delta {\rm{G}}}_{{\rm{U}}}-{\rm{T}}\Delta {\rm{S}}$$

where ΔE, ΔG_ZPE_, ΔG_U_, and ΔS refer to the DFT calculated energy change, the correction from zero-point energy, the correction from inner energy, and the correction from entropy^59^. The transition state was located via constrained optimization based on a process of varying the target reaction coordinate while relaxing all other degrees of freedom. The optimized structure was subsequently validated by a vibrational frequency analysis, confirming the presence of exactly only one imaginary frequency^60,61^.

### Reporting summary

Further information on research design is available in the Nature Portfolio Reporting Summary linked to this article.

## Supplementary information

Supplementary Information

Reporting Summary

Transparent Peer Review file

## Source data

Source Data

## Data Availability

The data generated in this study are provided in the Supplementary Information/Source Data file. Data are available from the corresponding authors upon request. Source data are provided with this paper.
