# Supplementary material for: Temperature-driven mechanistic transition in propylene oxidation over Pt/CeO2 ensemble catalysts
Source: Nat Commun. 2025 Oct 16;16:9199. doi: 10.1038/s41467-025-64243-y (PMC12532794; doi:10.1038/s41467-025-64243-y)
Supplement: Supplementary file 1 — Supplementary Information [file 41467_2025_64243_MOESM1_ESM.pdf]

Supplementary Information

Temperature-Driven Mechanistic Transition in Propylene Oxidation over Pt/CeO_2 Ensemble Catalysts

Zihao Li [1,2], Xingyan Chen [1], Yao Lv [3], Sheng Dai [3], Huazhen Chang [4], Zhenguo Li [5], Kailong Ye [6], Fudong Liu [6,*], Lei Ma [1,*], Naiqiang Yan [1]

[1] State Key Laboratory of Green Papermaking and Resource Recycling, School of Environmental Science and Engineering, Shanghai Jiao Tong University, Shanghai 200240, China

[2] PetroChina Petrochemical Research Institute, CNPC Company, Beijing 102206, China

[3] Key Laboratory for Advanced Materials and Feringa Nobel Prize Scientist Joint Research Center, School of Chemistry & Molecular Engineering, East China University of Science and Technology, Shanghai 200237, China

[4] School of Chemistry and Life Resources, Renmin University of China, Beijing 100872, China

[5] National Engineering Laboratory for Mobile Source Emission Control Technology, China Automotive Technology & Research Center Co., Ltd., Tianjin, 300300, China

[6] Department of Chemical and Environmental Engineering, Bourns College of Engineering, Center for Environmental Research and Technology (CE-CERT), Materials Science and Engineering (MSE) Program, UCR Center for Catalysis, University of California, Riverside, California 92521, United States

*Corresponding Authors: leima8@sjtu.edu.cn (L. Ma); fudong.liu@ucr.edu (F. Liu)

Supplementary Figures

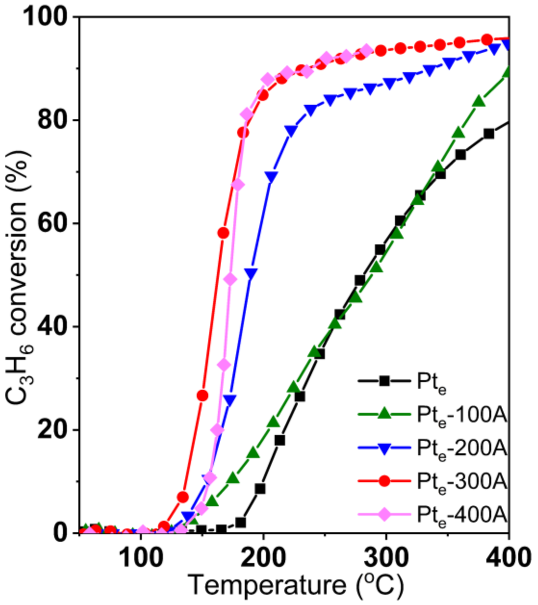

Supplementary Fig. 1 Catalytic activities of C_3H_6 oxidation over supported Pt catalysts pretreated under different temperatures. Reaction condition: 1000 ppm C_3H_6 and 10% O_2 in N_2 balance with a WHSV of 240, 000 mL g^{-1} h^{-1}.

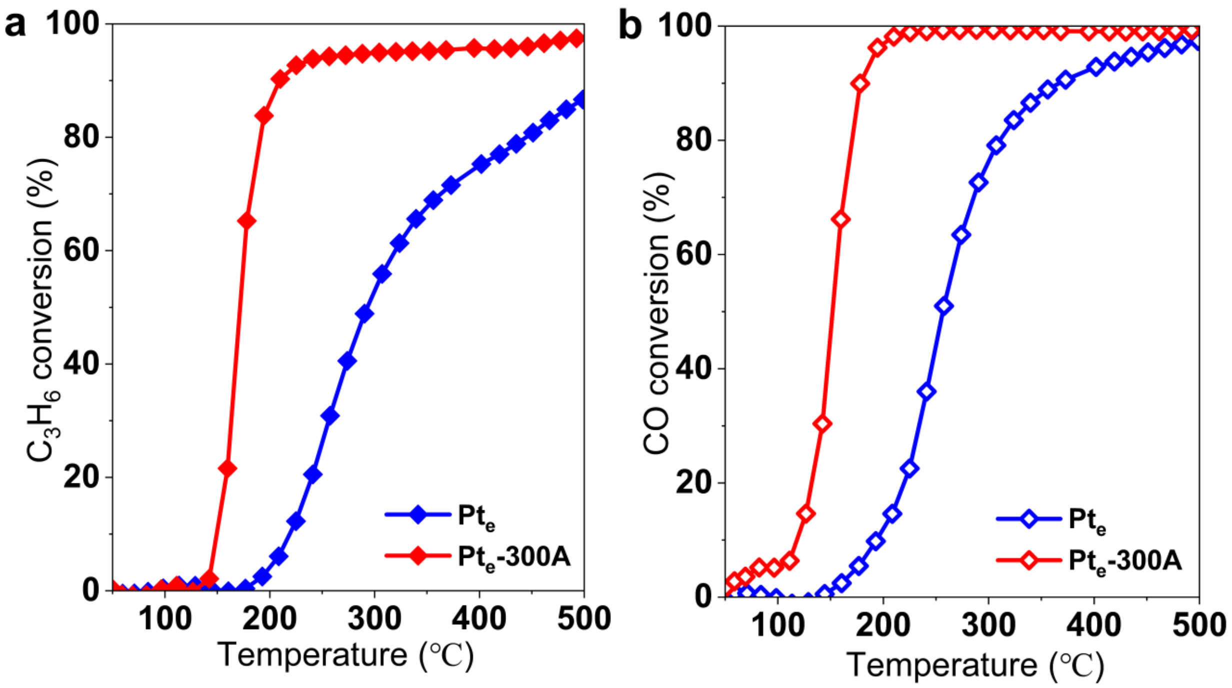

Supplementary Fig. 2 Catalytic activities of C_3H_6 and CO oxidation over supported Pt catalysts. a C_3H_6 and **b** CO co-oxidation light-off curves. Reaction condition: 1000 ppm C_3H_6, 4000 ppm CO, and 10% O_2 in N_2 balance with a WHSV of 240, 000 mL g^{-1} h^{-1}.

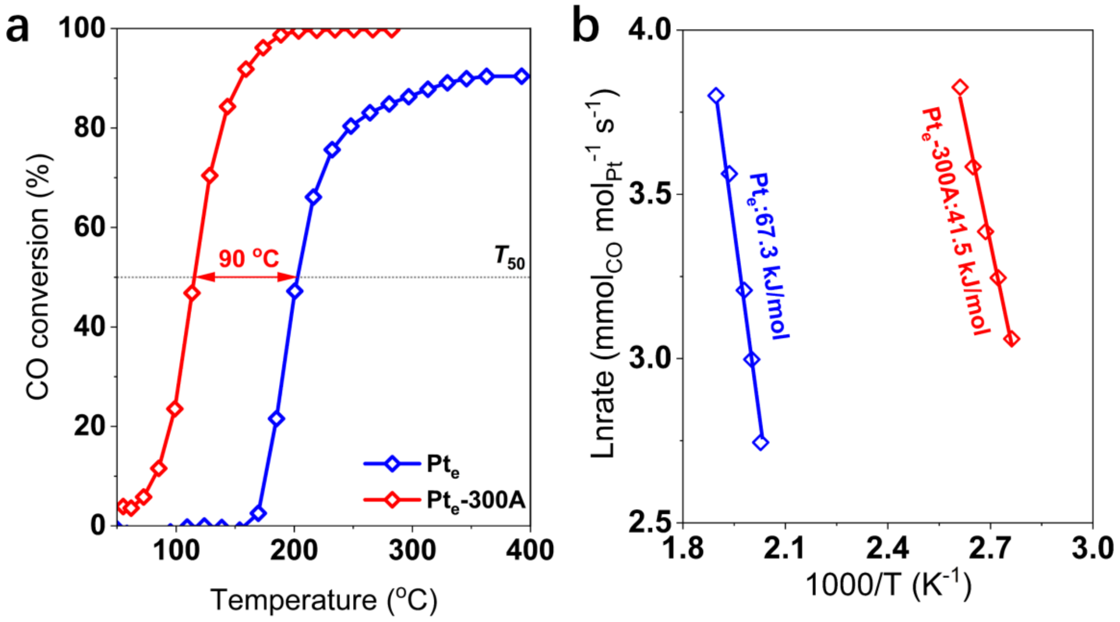

Supplementary Fig. 3 Catalytic activities and apparent activation energies of CO oxidation over supported Pt catalysts. a CO oxidation light-off curves; **b** Arrhenius plots of CO oxidation. Reaction condition: 4000 ppm CO and 10% O_2 in N_2 balance with a WHSV of 240, 000 mL g^{-1} h^{-1}.

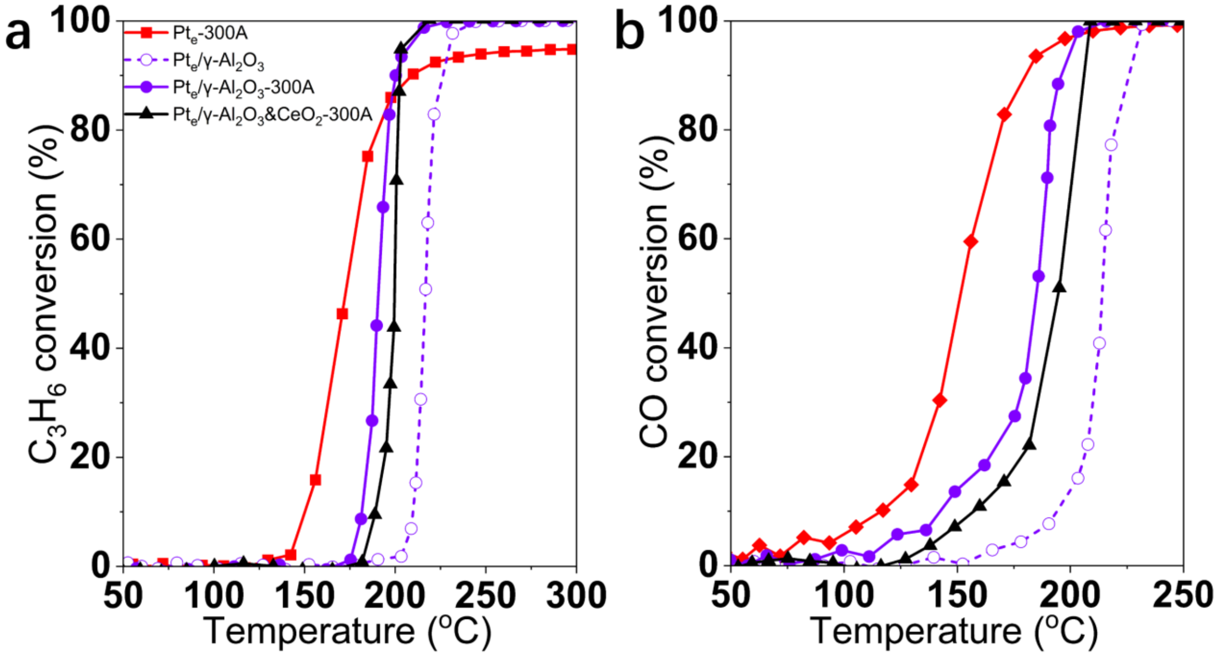

Supplementary Fig. 4 Catalytic activities of C_3H_6 and CO oxidation over Pt$_e$-300A and different Pt$_e$/γ-Al_2O_3 catalysts. **a** C_3H_6 and **b** CO oxidation light-off curves. Reaction condition: 1000 ppm C_3H_6, 4000 ppm CO, and 10% O_2 in N_2 balance with a WHSV of 240, 000 mL g^{-1} h^{-1}. Pt/γ-Al_2O_3&CeO_2-300A represented the physically mixed Pt/γ-Al_2O_3 and CeO_2, which was activated by H_2 at 300 °C.

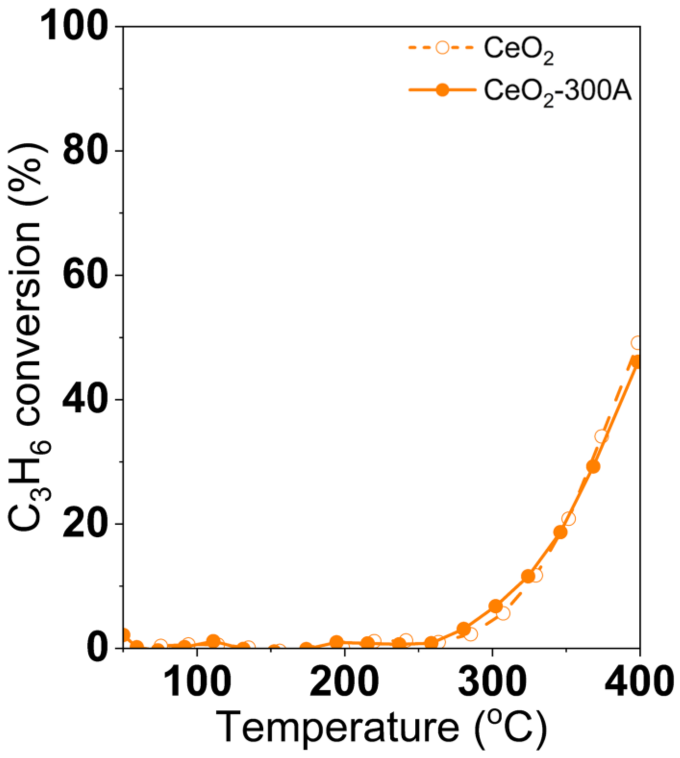

Supplementary Fig. 5 Catalytic activities of C_3H_6 oxidation over bare CeO_2 in the presence and absence of H_2 activation at 300 °C. Reaction condition: 1000 ppm C_3H_6, and 10% O_2 in N_2 balance with a WHSV of 240, 000 mL g^{-1} h^{-1}.

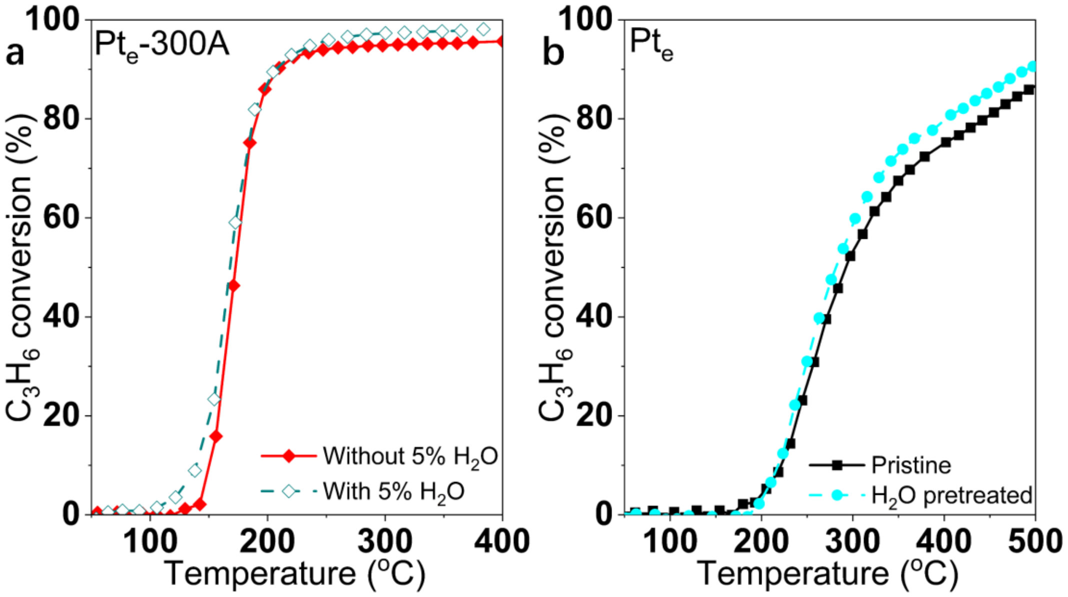

Supplementary Fig. 6 Analysis of water and surface hydroxyl influence on C_3H_6 oxidation activity. a C_3H_6 oxidation light-off performance in the presence and absence of 5% H_2O over Pt$_e$-300A. Reaction condition: 1000 ppm C_3H_6, 10% O_2, and 5% H_2O (when used) in N_2 balance with a WHSV of 240, 000 mL g^{-1} h^{-1}. **b** C_3H_6 oxidation light-off curves of Pt$_e$ in the presence or absence of H_2O pretreatment, respectively. The pretreatment was carried out under 10% H_2O/N_2 flow at 300 °C for 1 h. Reaction condition: 1000 ppm C_3H_6, and 10% O_2 in N_2 balance with a WHSV of 240, 000 mL g^{-1} h^{-1}.

Supplementary Note 1:

According to previous literature, Nie et al. indicated that the 10% H_2O pretreatment at 750 °C would cause the migration of oxygen vacancies from the bulk phase to the surface of Pt_1/CeO_2 catalysts, which would further couple with the H_2O to generate active surface -OH species and promoted the CO oxidation activity in the low-temperature regime [1]. As a result, the effect of water vapor on Pt$_e$-300A was directly tested and exhibited *via* C_3H_6 oxidation light-off, where water vapor did not affect the catalytic reactivity. Furthermore, even though Pt$_e$ catalysts were only activated in the H_2 atmosphere in the current investigation, exploring the probable contribution of surface -OH species to the catalytic oxidation activity, which could be produced during the H_2 prereduction, was critical. Therefore, the flow was switched from 10% H_2 to 10% H_2O at 300 °C for 1 h and cooled down in 10% H_2O/N_2 flow to room temperature to generate additional hydroxyl groups on the surface of Pt$_e$ catalysts.

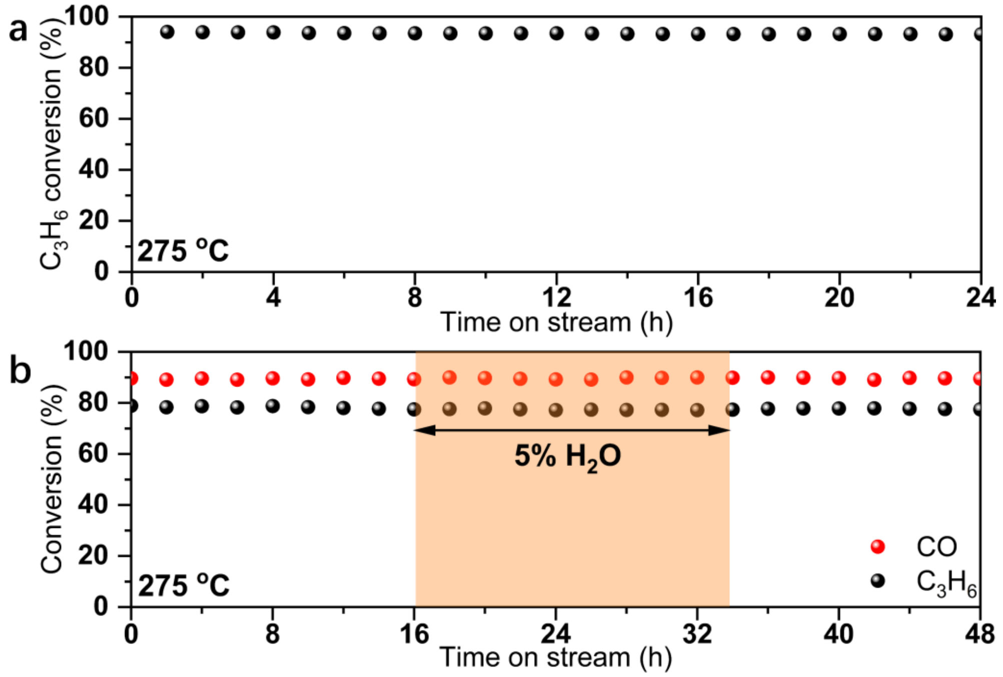

Supplementary Fig. 7 Stability tests over Pt$_e$-300A catalyst. a Thermal durability test of C_3H_6 oxidation at 275 °C. Reaction condition: 1000 ppm C_3H_6 and 10% O_2 in N_2 balance with a WHSV of 240, 000 mL g^{-1} h^{-1}. **b** Thermal durability test of C_3H_6 oxidation in the presence of H_2O at 275 °C. Reaction condition: 1000 ppm C_3H_6, 4000 ppm CO, 10% O_2, and 5% H_2O (when used) in N_2 balance with a WHSV of 240, 000 mL g^{-1} h^{-1}.

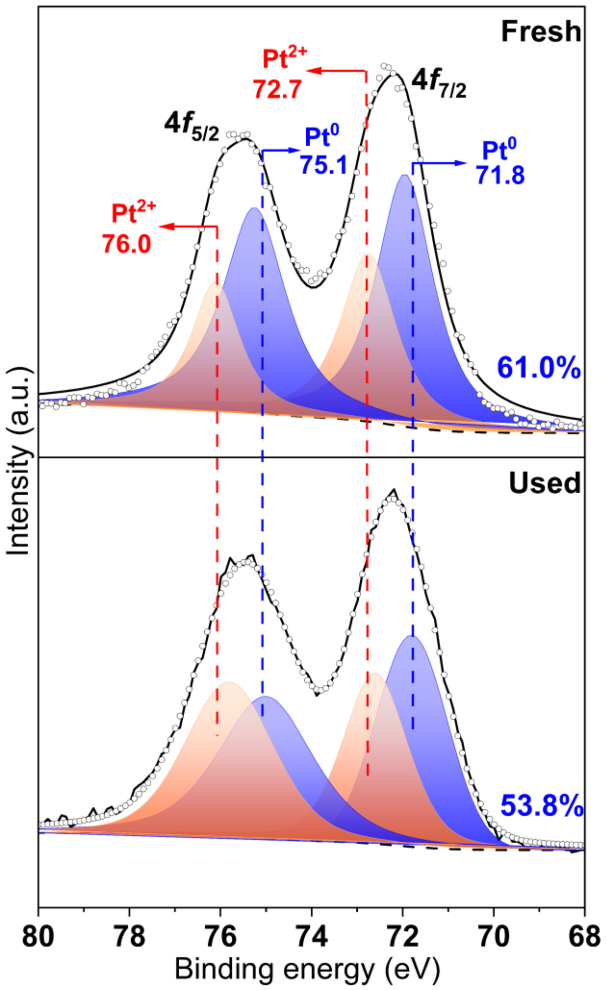

Supplementary Fig. 8 Pt 4*f* of XPS spectra for fresh and used Pt_e-300A catalysts. The used catalysts represent that fresh Pt_e-300A went through the thermal durability tests of C_3H_6 oxidation at 275 °C for 24 h. Reaction condition: 1000 ppm C_3H_6 and 10% O_2 in N_2 balance with a WHSV of 240, 000 mL g^{-1} h^{-1}.

Supplementary Note 2:

XPS experiments were performed to measure the chemical states of the supported Pt species by analyzing Pt 4*f*. The deconvoluted doublets were observed at 71.8 and 72.7 eV, corresponding to Pt^0 and Pt^{2+} in Pt $4f_{7/2}$ spectra, respectively. The Pt^0 ratio was slightly changed from 61.0% to 53.8%, indicating the relatively stable metallic Pt species during the catalytic tests under rich oxygen conditions.

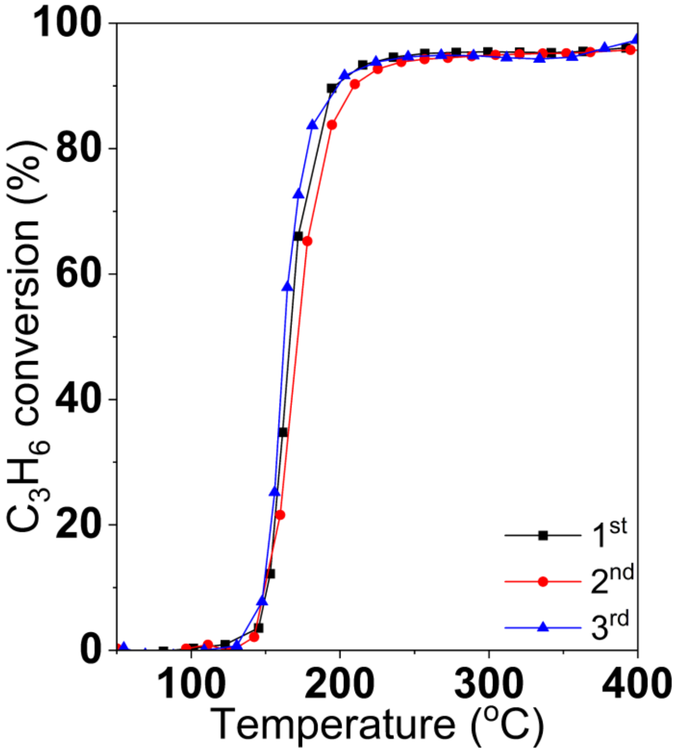

Supplementary Fig. 9 Cycling tests of the catalytic activities of C_3H_6 oxidation over Pt_e-300A catalyst. Reaction condition: 1000 ppm C_3H_6, 4000 ppm CO, and 10% O_2 in N_2 balance with a WHSV of 240, 000 mL g^{-1} h^{-1}.

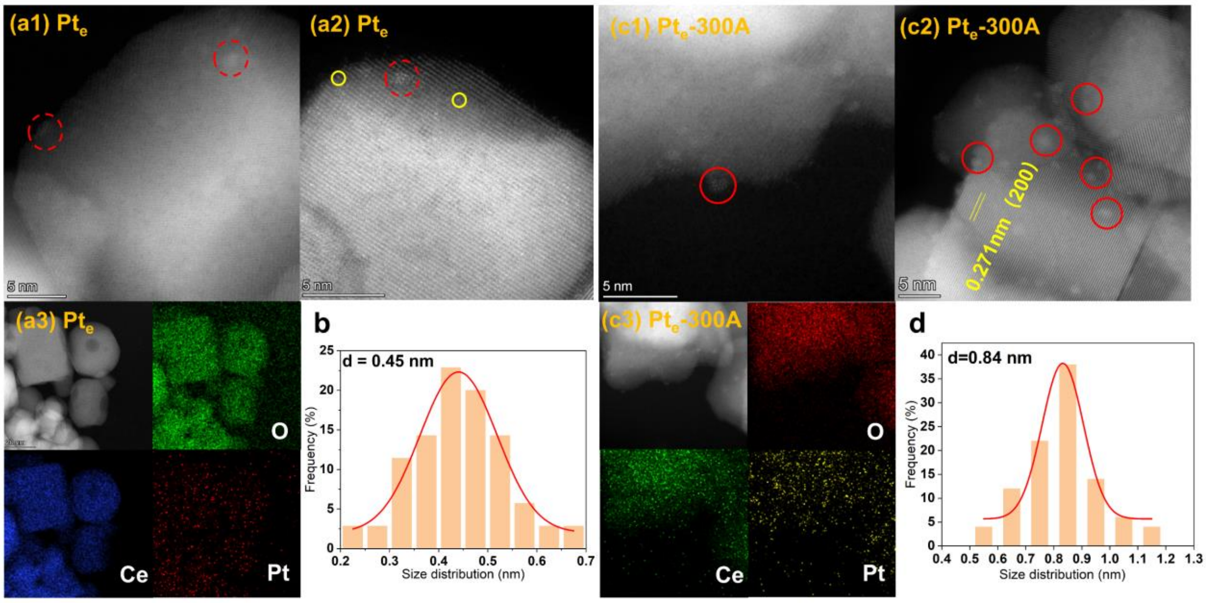

Supplementary Fig. 10 HAADF-STEM images and size distribution of Pt clusters over Pt$_e$ and Pt$_e$-300A catalysts. a, c Additional HAADF-STEM images and EDX-mapping of Pt$_e$ and Pt$_e$-300A, respectively (yellow cycle: Pt single atoms; red dashed cycle: planner single-layer Pt ensembles; red solid cycle: multilayer Pt ensembles); **b, d** size distribution of Pt nanoclusters over Pt$_e$ and Pt$_e$-300A, respectively.

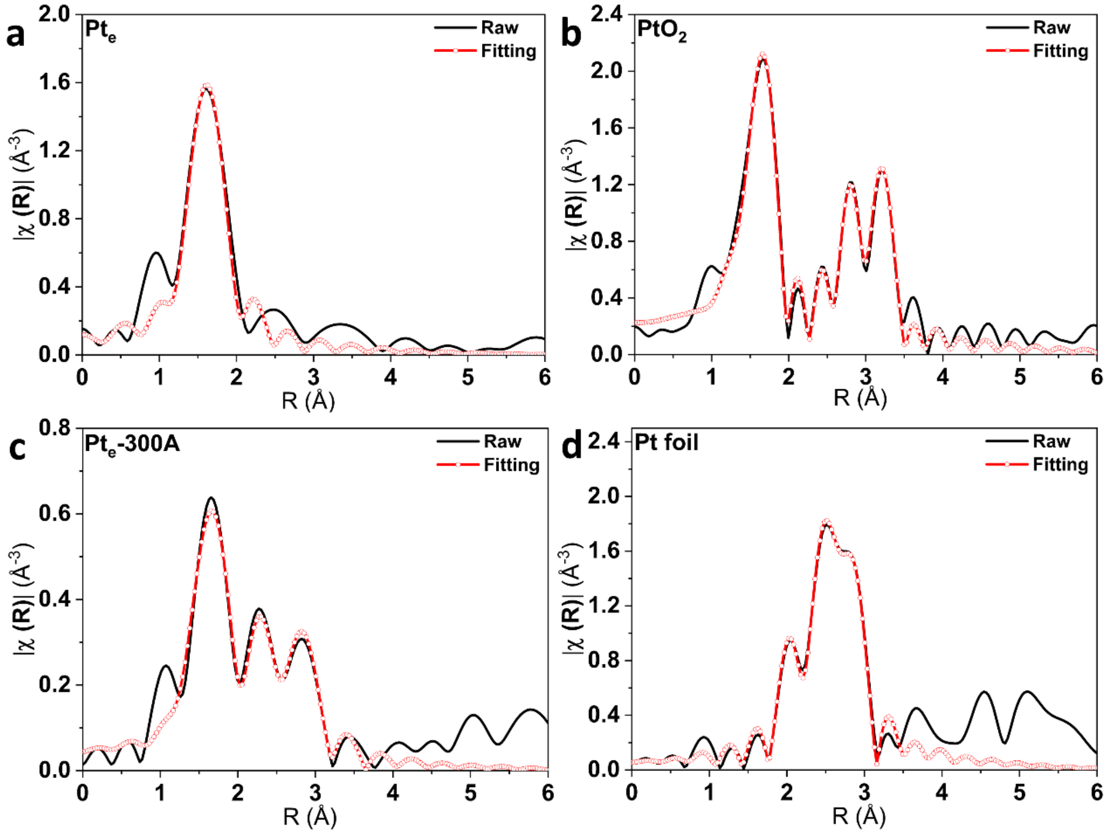

Supplementary Fig. 11 Fitting results of the Pt L_3-edge EXAFS spectra in R-space. **a** Pt_e; **b** PtO_2; **c** Pt_e-300A; **d** Pt foil (black solid: raw experimental data; red dotted: fitted data).

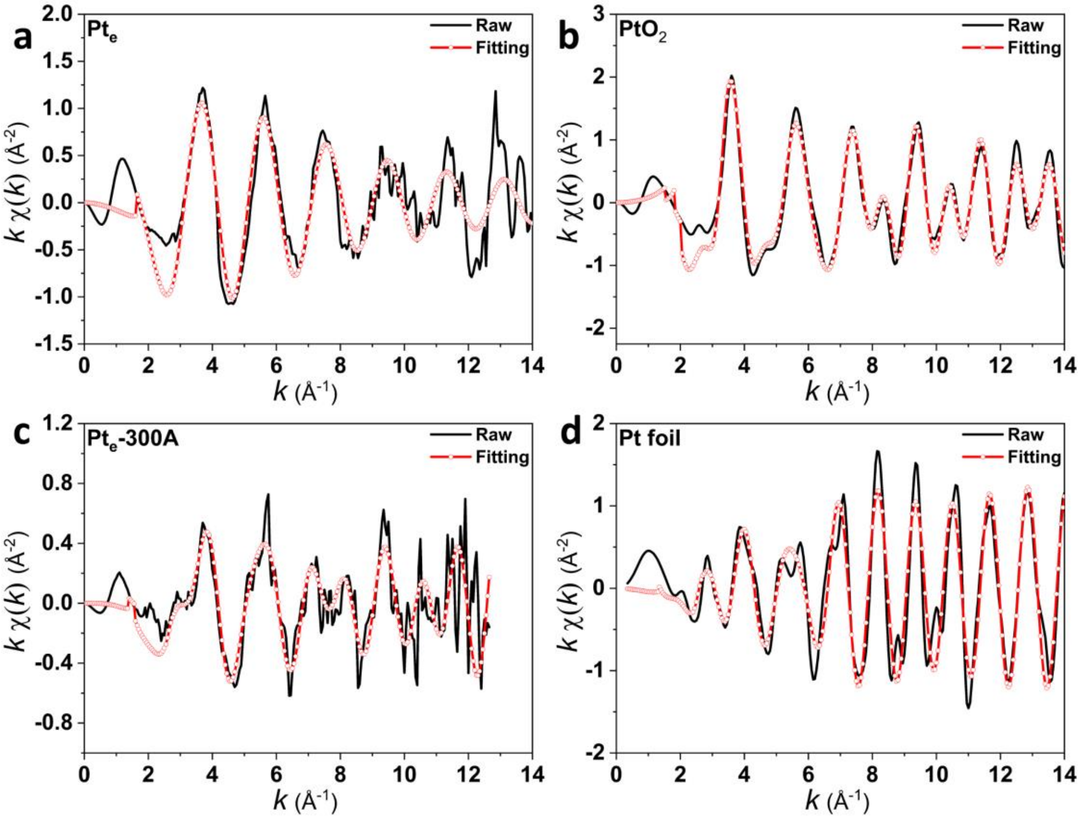

Supplementary Fig. 12 Fitting results of the Pt L_3-edge EXAFS spectra in *k*-space. a Pt_e; **b** PtO_2; **c** Pt_e-300A; **d** Pt foil (black solid: raw experimental data; red dotted: fitted data).

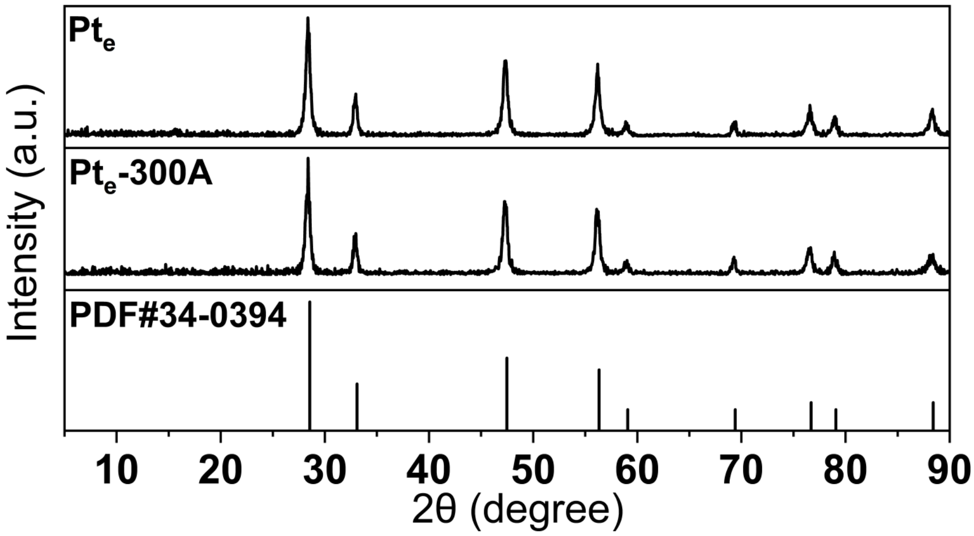

Supplementary Fig. 13 XRD patterns of Pt$_e$-300A and Pt$_e$ catalysts.

Supplementary Note 3:

The diffraction peaks of all the catalysts are identical to the standard reference patterns of the cubic fluorite CeO_2 (PDF#34-0394). The peaks at 28.6, 33.1, 47.5, 56.3, 59.1, 69.4, 76.7, 79.1, and 88.4° corresponded to the crystal planes of (111), (200), (220), (311), (222), (400), (331), (420), and (422), respectively. None of the crystal patterns of Pt species were detected among Pt$_e$-300A and Pt$_e$ catalysts. Meanwhile, the diameter of supporting CeO_2 of various catalysts was calculated *via* Scherrer's equation regarding the (111) crystal plane at 28.6°. Pt$_e$ and Pt$_e$-300A obtained similar average diameters of 15.3 and 15.2 nm, respectively. It revealed that H_2 reduction did not change the textural structure of the as-synthesized Pt$_e$ catalysts.

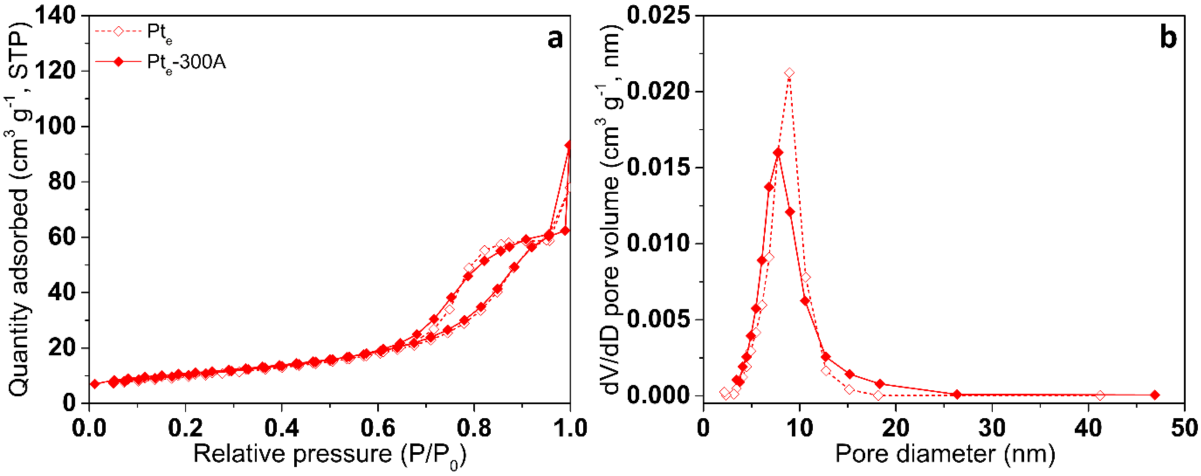

Supplementary Fig. 14 N_2 physisorption profiles. a Nitrogen adsorption-desorption isotherms and **b** BJH pore size distributions of Pt_e-300A and Pt_e samples.

Supplementary Note 4:

N_2 adsorption and desorption isotherms exhibited a type IV isotherm with H3 hysteresis loop [2], indicating the mesoporous structure on Pt_e-300A and Pt_e samples. The BET surface area was 38.4 and 36.3 $cm^2\ g^{-1}$ for Pt_e and Pt_e-300A, respectively, with similar pore volumes of 0.092 and 0.079 $cm^3\ g^{-1}$, indicating unchanged textural properties after H_2 activation.

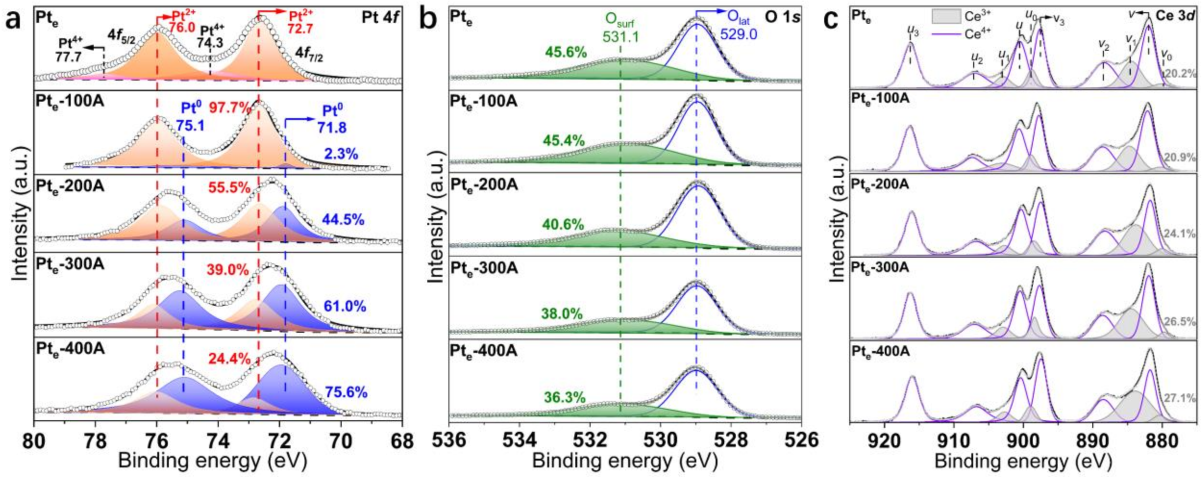

Supplementary Fig. 15 XPS for Pt_e-XA and Pt_e catalysts. a Pt 4*f*, **b** O 1*s*, and **c** Ce 3*d* regions.

Supplementary Note 5:

With H_2 activation, the Pt^0 ratio significantly increased from 0 to 61.0% for Pt_e and Pt_e-300A, respectively. At the same time, Ce^{3+} concentrations and surface-active oxygen species were not significantly changed. Two deconvolute peaks were presented on O 1*s* XPS profiles at approximately 531.2 and 528.9 eV, corresponding to the surface-active oxygen and lattice oxygen from CeO_2, respectively. The Ce 3*d* XPS profiles with Ce^{3+} and Ce^{4+} doublets were deconvoluted based on the initial states of $3d^{10}4f^1$ and $3d^{10}4f^{\circ}$ and various final states triggered by the transitions from valence band electrons to Ce 4*f* states [3,4]. After the H_2 activation, the concentration of Ce^{3+} increased and surface-active oxygen species declined with the rising reduction temperatures, respectively. Therefore, Ce^{3+} and surface-active oxygen species might not act as the dominant factors in the promotion of catalytic oxidation activity in comparison to the metallic Pt species. Meanwhile, since Pt_e-300A demonstrated the best low-temperature catalytic activity for both C_3H_6 and CO oxidation among Pt_e-XA samples (Supplementary Fig. 1), the optimal ratio between Pt^0 and Pt-O-Ce was expected to be 1.6.

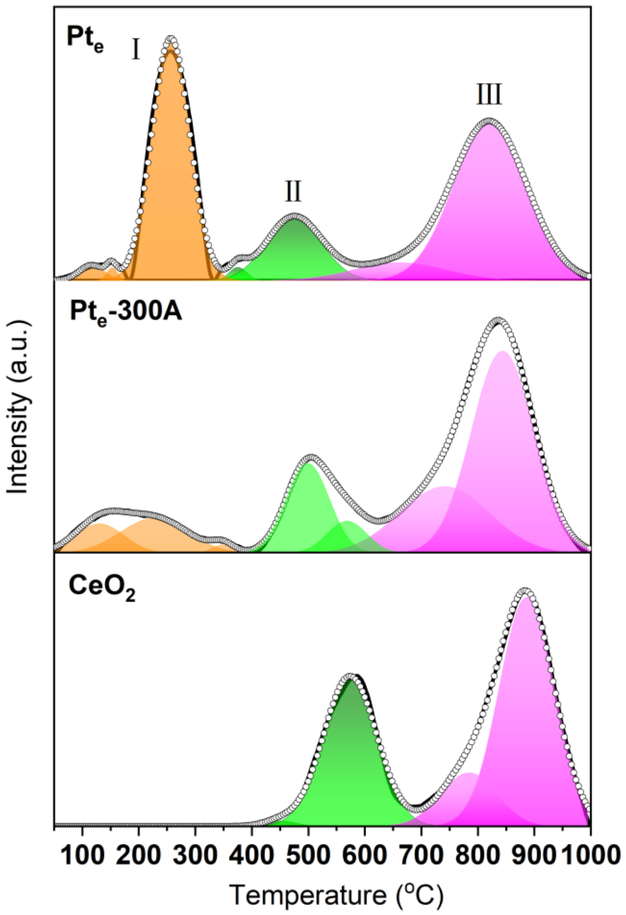

Supplementary Fig. 16 H_2-TPR profiles of CeO_2, Pt_e-300A, and Pt_e catalysts.

Supplementary Note 6:

The bare CeO_2 supports demonstrated two broad peaks (II and III) at approximately 580 and 884 °C, which could be ascribed to the reduction of the surface oxygen and CeO_2 to Ce_2O_3 by removing lattice oxygen, respectively [5]. With the loading of ensemble Pt nanoclusters, peaks II and III shifted to a lower temperature at Pt_e and Pt_e-300A catalysts, indicating improved reducibility due to the metal-supporting interaction. Meanwhile, the appearance of the reduction peak I below 260 °C could be deconvoluted into two doublets at approximately 145 and 250 °C, which could be attributed to the reduction of Pt-O and Pt-O-Ce, respectively [6]. H_2 reduction at 300 °C caused a significant declination of the relative area of peak I, indicating the formation of the metallic Pt with a small amount of PtO_x species remaining at the surface of CeO_2.

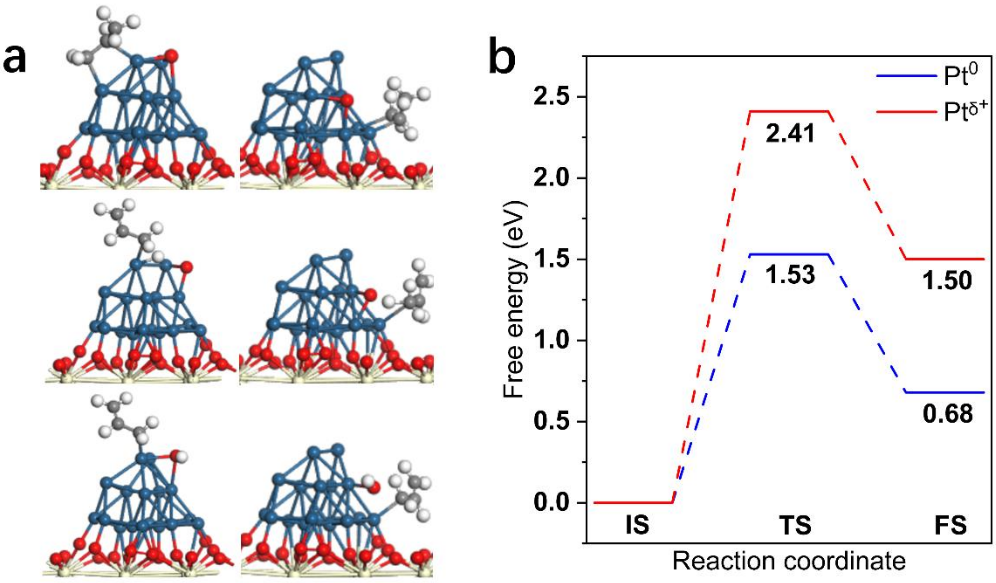

Supplementary Fig. 17 DFT calculations of dehydrogenation of methyl group on C_3H_6 at different Pt sites. a DFT calculated optimized structures of the initial state (IS), transition state (TS), final state (FS) (Grey: carbon; White: hydrogen; Blue: Pt atoms; Red: oxygen; Pale yellow: cerium); **b** Energy barriers of oxygen-facilitated dehydrogenation on Pt^0 and $Pt^{\delta+}$ sites.

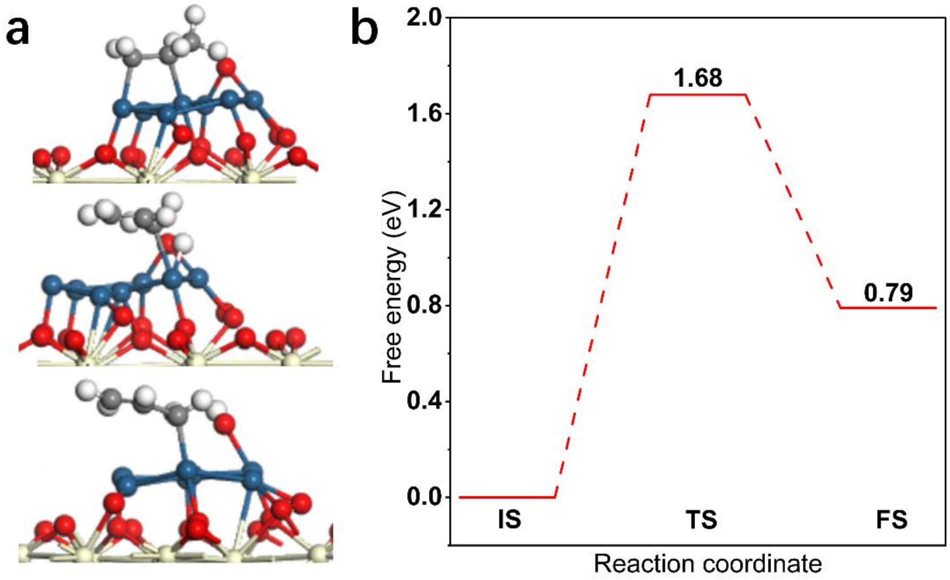

Supplementary Fig. 18 DFT calculations of dehydrogenation of methyl group in C_3H_6 at the as-synthesized, supported Pt catalysts. a DFT calculated optimized structures of the initial state (IS), transition state (TS), final state (FS) (Grey: carbon; White: hydrogen; Blue: Pt atoms; Red: oxygen; Pale yellow: cerium); **b** Energy barriers of oxygen-facilitated dehydrogenation on single-layer Pt ensemble.

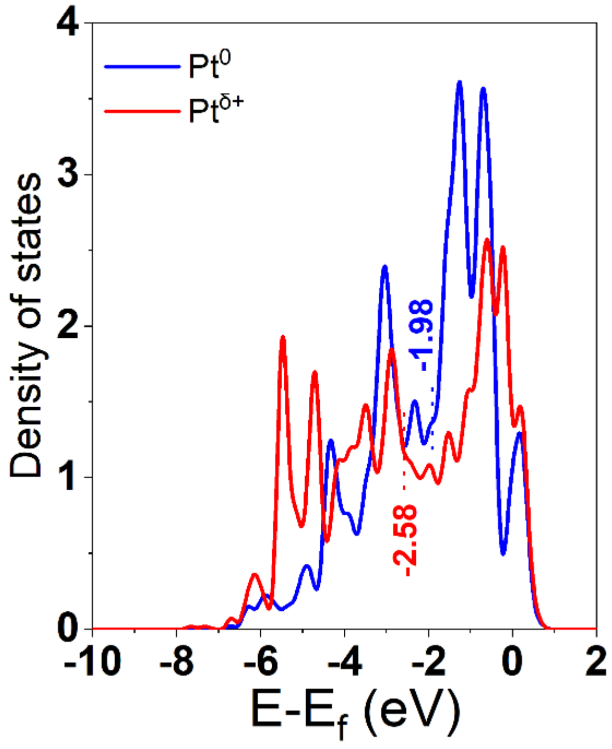

Supplementary Fig. 19 Density of states projected on the 5d-orbital of Pt for Pt^0 and Pt$^{\delta+}$ sites. The indicated number represents the d-band center for 5d-orbital of Pt.

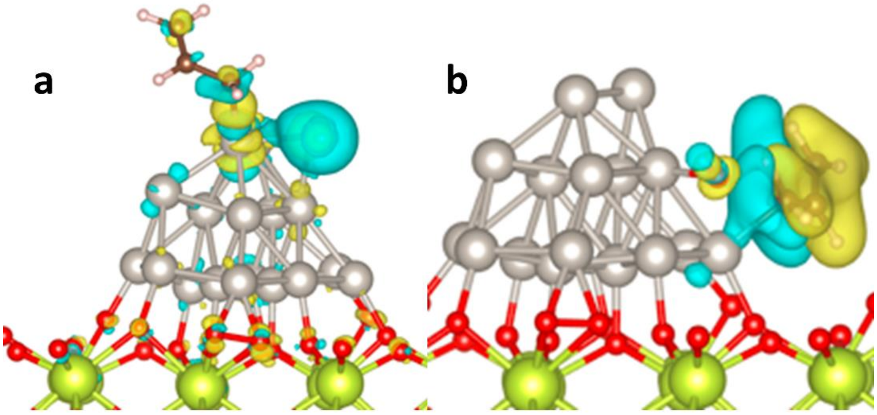

Supplementary Fig. 20 The difference charge density of the oxygen-facilitated dehydrogenation of sp^3 hybrid carbon over Pt$_e$-300A. a Pt^0 ensemble sites and **b** Pt$^{\delta+}$ bottom single-layer sites. The charge density of blue and yellow represents the charge consumption and charge accumulation, respectively.

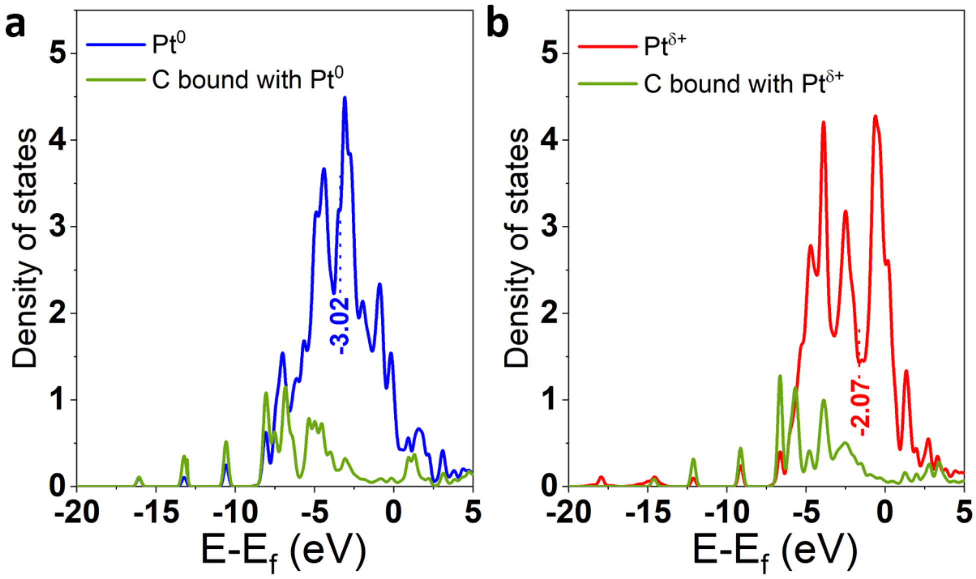

Supplementary Fig. 21 Density of states projected on the 5d-orbital of Pt and 2p-orbital of C for C_3H_6 adsorption at a Pt^0 and **b** $Pt^{\delta+}$ sites. The indicated number represents the d-band center for 5d-orbital of Pt.

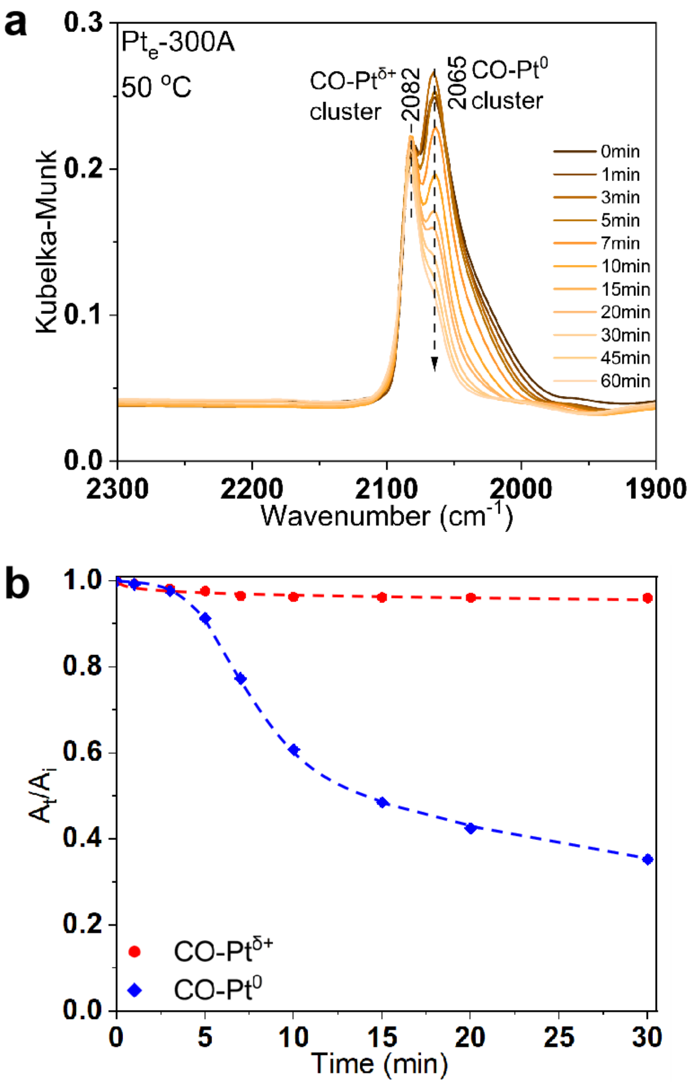

Supplementary Fig. 22 Investigation of CO oxidation activity over Pt^0 and Pt$^{\delta+}$ sites. a *in situ* DRIFTs spectra of the reaction between preabsorbed CO and purging O_2 over Pt$_e$-300A at 50 °C. **b** Normalized peaks area of CO adsorption on Pt^0 and Pt$^{\delta+}$ sites as a function of time at 50 °C.

Supplementary Note 7:

Post CO adsorption and N_2 purge, Pt$_e$-300A catalysts displayed two intensive bands at approximately 2082 and 2065 cm^{-1}, which could be assigned to the CO linearly adsorbed on Pt$^{\delta+}$ and Pt^0 sites, respectively. To quantitatively compare the inherent reactivity over the different Pt sites, the reaction rate was calculated and plotted by integrating the area of peaks for CO-Pt^0@Pt$_e$ and CO-Pt$^{\delta+}$@Pt$_e$ as a function of reaction time. At 50 °C, CO-Pt$^{\delta+}$@Pt$_e$ barely reacted with O_2. However, CO was rapidly consumed by O_2 on Pt^0 sites, indicating a much better reactivity on Pt^0 than Pt$^{\delta+}$ sites.

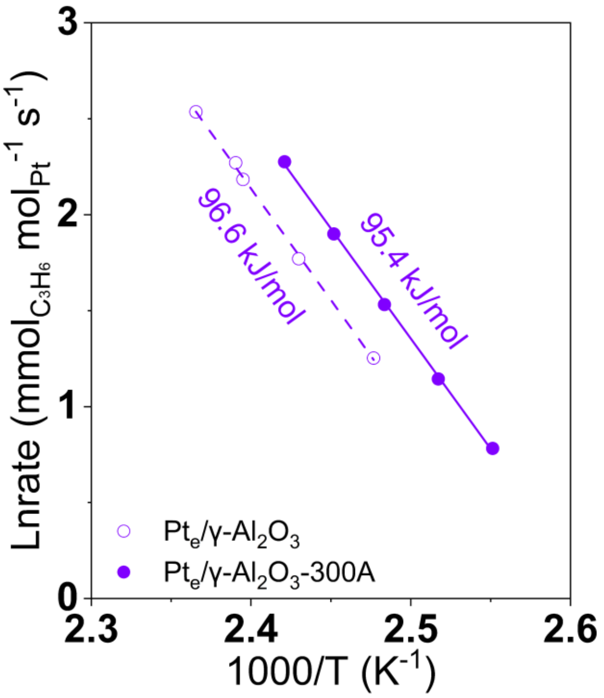

Supplementary Fig. 23 Arrhenius plots of C_3H_6 oxidation over Pt_e/γ-Al_2O_3 and Pt_e/γ-Al_2O_3-300A catalysts. Reaction condition: 1000 ppm C_3H_6, and 10% O_2 in N_2 balance with a WHSV of 240, 000 mL g^{-1} h^{-1}.

Supplementary Note 8:

H_2 activation over Pt_e/γ-Al_2O_3 also improved the catalytic performance for C_3H_6 oxidation. However, Pt_e/γ-Al_2O_3 and Pt_e/γ-Al_2O_3-300A samples had similar apparent activation energies for the oxidation of C_3H_6, indicating that the fresh and H_2-activated catalysts exhibited the same active sites.

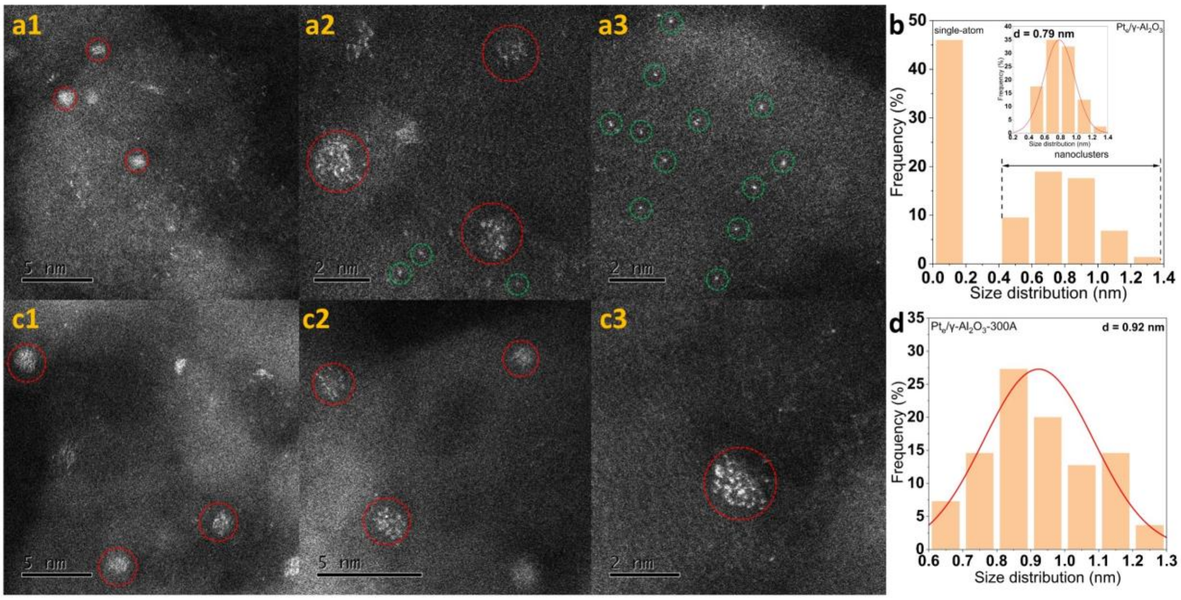

Supplementary Fig. 24 HAADF-STEM images and size distribution of Pt clusters over Pt_e/γ-Al_2O_3 and Pt_e/γ-Al_2O_3-300A catalysts. a, c Additional HAADF-STEM images of Pt_e/γ-Al_2O_3 and Pt_e/γ-Al_2O_3-300A catalysts, respectively (red cycle: Pt nanocluster; green cycle: Pt single atom); **b, d** size distribution of Pt ensembles over Pt_e/γ-Al_2O_3 and Pt_e/γ-Al_2O_3-300A catalysts, respectively.

Supplementary Note 9:

The morphology of Pt species over Pt_e/γ-Al_2O_3 and Pt_e/γ-Al_2O_3-300A catalysts was also captured and demonstrated. For Pt_e/γ-Al_2O_3 sample, Pt ensembles existed with a mean diameter of approximately 0.79 nm. Meanwhile, abundant Pt single atoms, with approximately 46%, could also be detected at the surface of Pt_e/γ-Al_2O_3 catalyst. The number of Pt single atoms was reduced dramatically after H_2 activation, accompanied by a significant rise in the average diameter of Pt ensembles (0.92 nm), which might be triggered by the migration of the Pt single atoms to the neighboring ensembles.

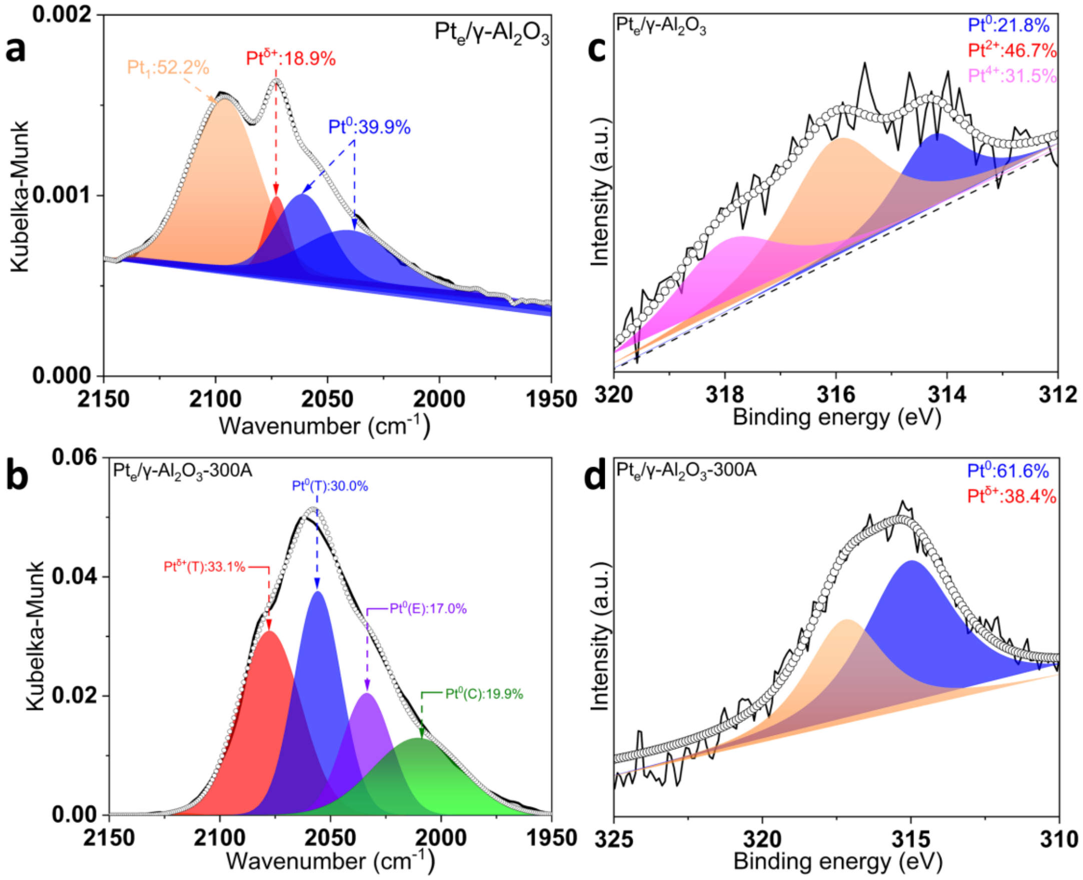

Supplementary Fig. 25 Spectroscopic assignment of Pt^0 and Pt$^{δ+}$ over Pt$_e$/γ-Al$_2$O$_3$ and Pt$_e$/γ-Al$_2$O$_3$-300A catalysts. a, b *in situ* DRIFTs of CO adsorption on Pt$_e$/γ-Al$_2$O$_3$ and Pt$_e$/γ-Al$_2$O$_3$-300A, respectively. **c, d** XPS spectra of Pt 4*d* on Pt$_e$/γ-Al$_2$O$_3$ and Pt$_e$/γ-Al$_2$O$_3$-300A, respectively.

Supplementary Note 10:

The as-synthesized Pt$_e$/γ-Al$_2$O$_3$ samples were mainly composed of the single atomic Pt at 2097 cm^{-1} and the Pt ensembles in the range of 2030-2085 cm^{-1} [7]. Since the Pt 4*f* overlapped with the Al 2*p*, Pt 4*d* spectra were measured over Pt$_e$/γ-Al$_2$O$_3$ and Pt$_e$/γ-Al$_2$O$_3$-300A catalysts to determine the chemical valence of Pt species. Different from the Pt$_e$, fresh Pt$_e$/γ-Al$_2$O$_3$ catalysts already possessed approximately 21.8% Pt^0 regarding the Pt 4*d* XPS spectra, which was similar to the *in situ* DRIFTs integration ratio (19.8%). In addition to significantly increasing the metallic Pt ratio in the XPS spectra to 61.6%, the H_2 reduction activation also sparked the assembly of single atoms Pt, based on the disappearance of the IR band at around 2100 cm^{-1}, into larger ensembles with a higher amount of Pt^0 sites (66.9%). Based on the XPS and *in situ* DRIFTs results mentioned above, the increasing number of Pt^0 sites for Pt$_e$/γ-Al$_2$O$_3$ catalysts

after the H_2 reduction activation prompted the promotion of the catalytic oxidation performance, reinforcing the universality of the metallic Pt as the intrinsic active sites for the low-temperature oxidation.

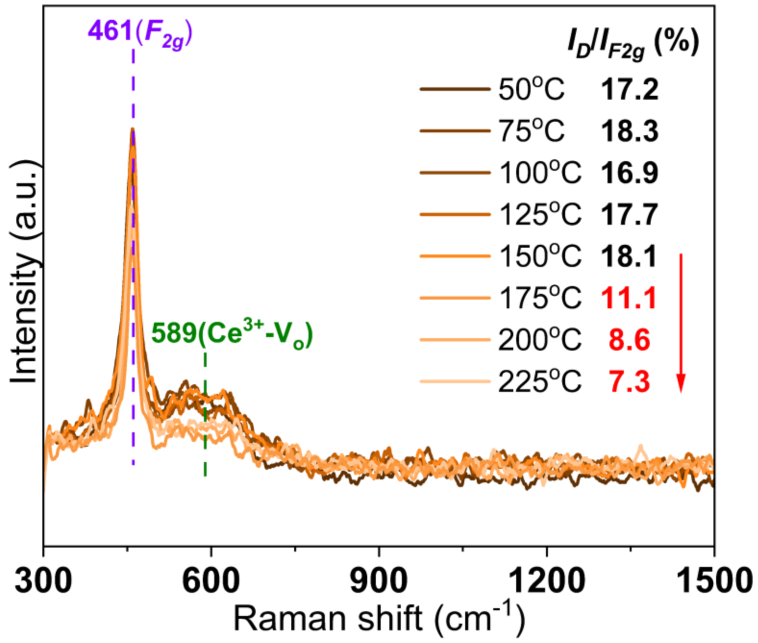

Supplementary Fig. 26 Operando Raman spectra of Pt_e-300A under CO oxidation from 50 to 225 °C.

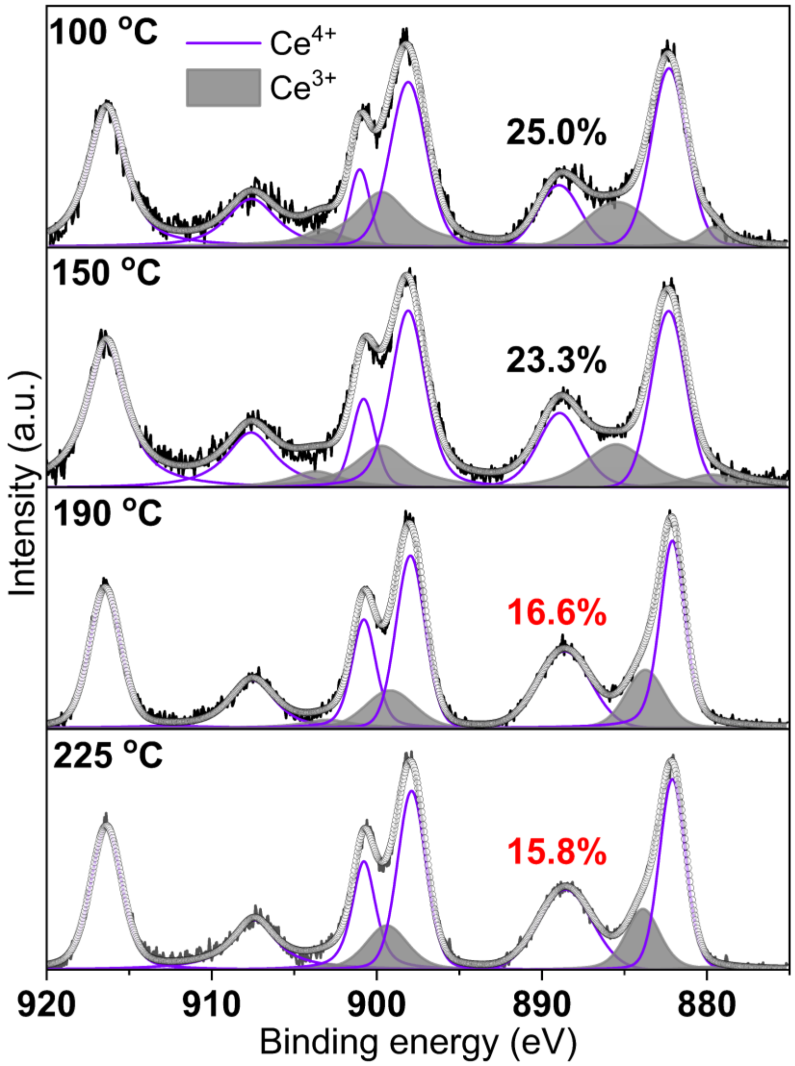

Supplementary Fig. 27 NAP-XPS of Ce 3*d* over Pt$_e$-300A during C_3H_6 oxidation at different temperatures.

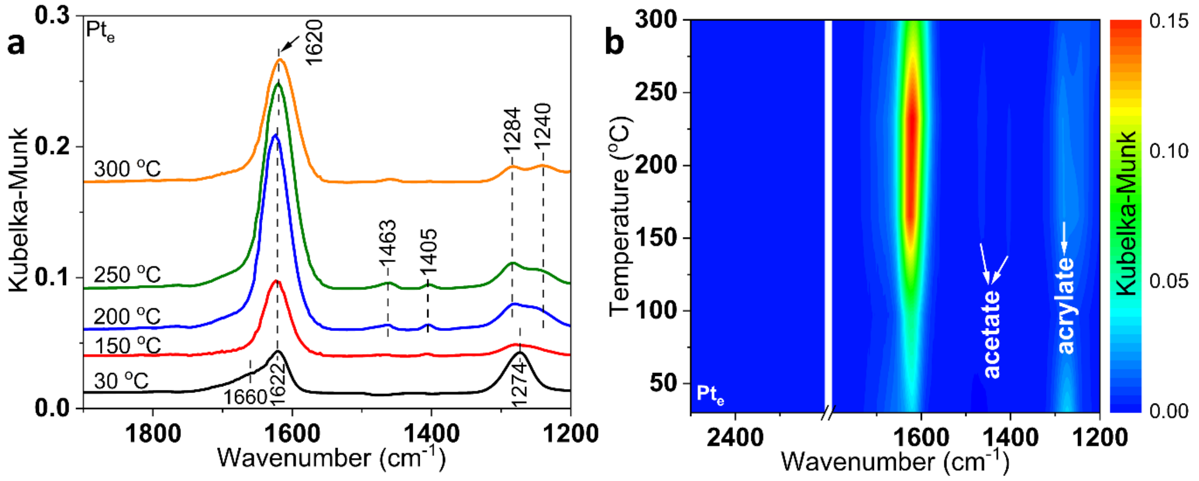

Supplementary Fig. 28 DRIFTs analysis of C_3H_6 oxidation over Pt$_e$. a *in situ* DRIFTs spectra of steady-state C_3H_6 and O_2 co-adsorption; **b** Contour graphs for C_3H_6 oxidation.

1. $O_2 + * \overset{K_1}{\rightleftharpoons} O_2*$
2. $O_2* + * \overset{K_2}{\rightleftharpoons} 2O*$
3. $C_3H_6 + * \overset{K_3}{\rightleftharpoons} C_3H_6*$
4. $C_3H_6* + O* \xrightarrow{k_4} C_3H_5* + OH*$
5. $C_3H_5* + 11O* \overset{K_5}{\rightleftharpoons} 3CO_2* + 5OH* + 4*$
6. $CO_2* \overset{K_6}{\rightleftharpoons} CO_2 + *$
7. $OH* + OH* \overset{K_7}{\rightleftharpoons} H_2O* + O*$
8. $H_2O* \overset{K_8}{\rightleftharpoons} H_2O + *$

Supplementary Fig. 29 The elementary step of C_3H_6 oxidation at 162 ^oC. * is the unoccupied surface vacant sites over Pt metals; k and K are rate constant and equilibrium constant, respectively.

Supplementary Note 11:

The assumption of pseudo-steady state for all the adsorbed species and quasi-equilibrium for step 1, 2, and 3 leads to the rate equation of C_3H_6 oxidation (Supplementary Equation 1).

$$r = K_3 k_4 [C_3H_6] \sqrt{K_1 K_2 [O_2]} \frac{1}{(1 + K_1[O_2] + \sqrt{K_1 K_2 [O_2]} + K_3[C_3H_6])^2} \quad (1)$$

1. $O_2 + \# \overset{K_1}{\rightleftharpoons} O_2^{\#}$

2. $O_2^{\#} + \# \overset{K_2}{\rightleftharpoons} 2O^{\#}$

3. $C_3H_6 + * \overset{K_3}{\rightleftharpoons} C_3H_6*$

4. $C_3H_6* + O^{\#} \xrightarrow{k_4} C_3H_5* + OH^{\#}$

5. $C_3H_5* + 11O^{\#} \overset{K_5}{\rightleftharpoons} 3CO_2* + 5OH^{\#} + * + 6\#$

6. $CO_2* \overset{K_6}{\rightleftharpoons} CO_2 + *$

7. $OH^{\#} + OH^{\#} \overset{K_7}{\rightleftharpoons} H_2O^{\#} + O^{\#}$

8. $H_2O^{\#} \overset{K_8}{\rightleftharpoons} H_2O + \#$

Supplementary Fig. 30 The elementary step of C_3H_6 oxidation at 188 ^oC. * is the unoccupied surface vacant sites over Pt metals; # is the unoccupied vacant sites over Pt-O-Ce interfaces; k and K are rate constant and equilibrium constant, respectively.

Supplementary Note 12:

It could consider another Langmuir-Hinshelwood model at high reaction temperatures. In this model, it was speculated that C_3H_6 adsorbed and activated over the metallic Pt surface, where O_2 was adsorbed and activated over vacant sites on the Pt-O-Ce interfaces, without competition with C_3H_6. The assumption of pseudo-steady state for all the adsorbed species and quasi-equilibrium for step 1, 2, and 3 leads to the rate equation of C_3H_6 oxidation (Supplementary Equation 2). Considering the zero-order of C_3H_6 partial pressure, C_3H_6* could be the dominant surface species without any obvious O_2* and $O*$ on the surface. Therefore, the rate equation could be further simplified into Supplementary Equation 3, which was also in line with the kinetic data.

$$r = k_4 \frac{K_3[C_3H_6]}{1+K_3[C_3H_6]} \bullet \frac{\sqrt{K_1K_2[O_2]}}{1+K_1[O_2]+\sqrt{K_1K_2[O_2]}} \qquad (2)$$

$$r = k_4\sqrt{K_1K_2[O_2]} \qquad (3)$$

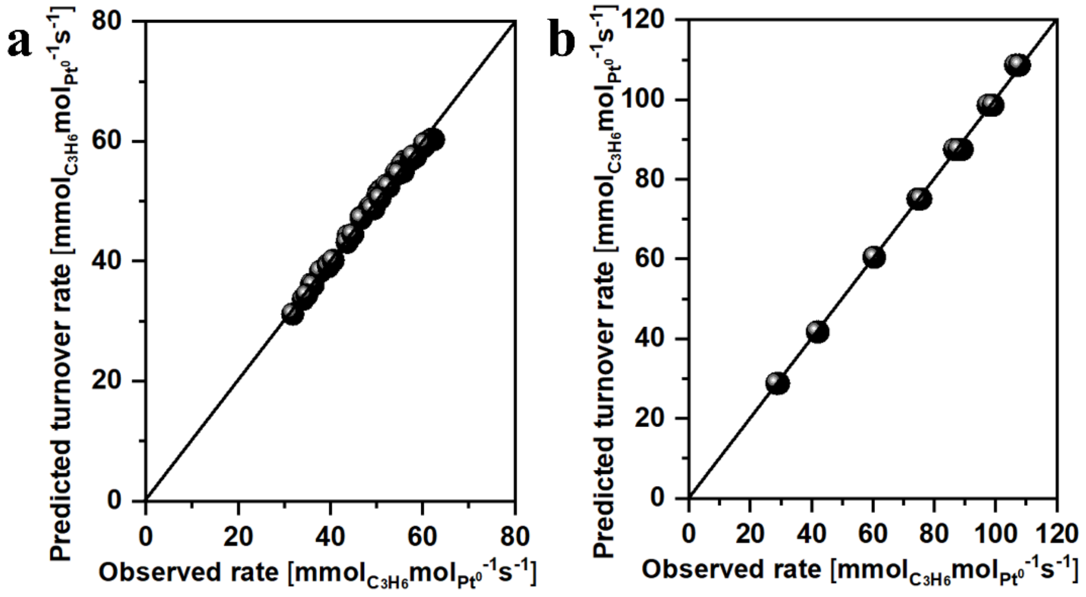

Supplementary Fig. 31 Parity plot of measured and predicted C_3H_6 turnover rates on Pt_e-300A catalysts at different reaction temperatures. a 162 °C and **b** 188 °C.

Supplementary Tables

Supplementary Table 1 Comparison of the reactivity of C_3H_6 oxidation between Pt_e-300A and previously reported Pt-based catalysts.

Samples	Reaction Conditions	C_3H_6 Consumption Rate ($mmol\cdot mol_{Pt}^{-1}\cdot s^{-1}$) [a]	T_{50} (°C)	Ref.
Pt@TiO_2 (0.89 wt.%)	[C_3H_6] = 800 ppm, [O_2] = 2 %, N_2 balance; WHSV=300,000 mL g^{-1} h^{-1}	3.12	225	8
Pt@TiSi (0.86 wt.%)	[C_3H_6] = 800 ppm, [O_2] = 2 %, N_2 balance; WHSV=300,000 mL g^{-1} h^{-1}	6.29	197	9
Pt/TiO_2 (0.5 wt.%)	[C_3H_6] = 10000 ppm, [O_2] = 20 %, N_2 balance; WHSV=60,000 mL g^{-1} h^{-1}	8.55	176	10
Pt/BaO/Al_2O_3 (2.0 wt.%)	[C_3H_6] = 800 ppm, [O_2] = 2 %, N_2 balance; WHSV=600,000 mL g^{-1} h^{-1}	13.42	210	11
Pt/Al_2O_3 (1.84 wt.%)	[C_3H_6]=1500 ppm, [O_2]=3%, [H_2O]=10%, Ar balance; WHSV=100,000 mL g^{-1} h^{-1}	17.95	122	12
Pt/Silicalite-1 (1.0 wt.%)	[C_3H_6] = 1000 ppm, [O_2] = 20 %, N_2 balance; WHSV=30,000 mL g^{-1} h^{-1}	6.63	112	13
PtC/Al (0.98 wt.%)	[C_3H_6] = 1000 ppm, [O_2] = 10 %, N_2 balance; WHSV=240,000 mL g^{-1} h^{-1}	54.17	180	14
PtY/Al (0.99 wt.%)	[C_3H_6] = 1000 ppm, [O_2] = 10 %, N_2 balance; WHSV=240,000 mL g^{-1} h^{-1}	25.20	201	14
Pt/CeO_2-a (1.0 wt.%)	[C_3H_6] = 2000 ppm, [O_2] = 2 %, Ar balance; WHSV=200,000 mL g^{-1} h^{-1}	17.69	223	15
Pt_e/CeO_2-300A (0.628 wt.%)	[C_3H_6] = 1000 ppm, [O_2] = 10 %, N_2 balance; WHSV=240,000 mL g^{-1} h^{-1}	71.85	162	This work

[a] The C_3H_6 consumption rate was calculated at the reaction temperature at 200 °C.

Supplementary Table 2 Comparison of the reactivity of CO oxidation between Pt_e-300A and previously reported Pt/CeO_2 catalysts.

Samples		Reaction Conditions	CO Consumption Rate ($mmol \cdot mol_{Pt}^{-1} \cdot s^{-1}$) [a]	Ref.
**Pt single atoms**	Pt/CeO_2_S (1.0 wt.%)	[CO] = 0.4 %, [O_2] = 10 % in Ar balance; WHSV=200,000 mL g^{-1} h^{-1}	58.91	[1]
	Pt_1/CeO_2_TS (1.0 wt.%)	[CO] = 1 %, [O_2] = 10 % in N_2 balance; WHSV=200,000 mL g^{-1} h^{-1}	64.96	[16]
	Pt/CeO_2-550 (0.92 wt.%)	[CO] = 1 %, [O_2] = 1 % in Ar balance; WHSV=400,000 mL g^{-1} h^{-1}	44.28	[17]
	$Pt_1/Ce_{0.99}Cu_{0.01}O_2$ (1.51 wt.%)	[CO] = 1 %, [O_2] = 10 % in N_2 balance; WHSV=120,000 mL g^{-1} h^{-1}	168.16	[18]
**Pt clusters**	Pt-O-Pt/CeO_2-a (2.8 wt.%)	[CO] = 0.1 %, [O_2] = 5 % in N_2 balance; WHSV=2,400,000 mL g^{-1} h^{-1}	110.03	[19]
	Pt_{ASL}/CA (0.25 wt.%)	[CO] = 1 %, [O_2] = 1 % in Ar balance; WHSV=200,000 mL g^{-1} h^{-1}	237.99	[20]
	2Pt/1Pt@CeO_2 (3 wt.%)	[CO] = 1.9 %, [O_2] = 1.3 % in N_2 balance; WHSV=232,500 mL g^{-1} h^{-1}	148.07	[21]
	Pt_e/CeO_2-300A (0.628 wt.%)	[CO] = 0.4 %, [O_2] = 10 % in N_2 balance; WHSV=240,000 mL g^{-1} h^{-1}	221.80	This work
**Pt particles**	Pt/CeO_2 (1.0 wt.%)	[CO] = 0.6 %, [O_2] = 0.6 % in N_2 balance; WHSV=80,000 mL g^{-1} h^{-1}	226.29	[22]
	Pt/CeO_2 TAPN (1.0 wt.%)	[CO] = 1.9 %, [O_2] = 1.3 % in He balance; WHSV=232,500 mL g^{-1} h^{-1}	99.88	[23]

[a] The CO consumption rate was calculated at the reaction temperature at 125 ^{o}C.

Supplementary Table 3 Relative area ratios of Ce^{3+}, O_{surf}, and Pt species from the deconvolution curves for Ce 3*d*, O 1*s*, and Pt 4*f* XPS spectra.

Samples	Ce^{3+} (%) [a]	$O_{surf}/[O_{lat}+O_{surf}]$ (%)	Pt^0 (%)	Pt^{2+} (%)	Pt^{4+} (%)
Pt_e	20.2	36.3	-	78.9	21.1
Pt_e-300A	26.5	45.4	61.0	39.0	-

[a] The concentration of Ce^{3+} was calculated by the equation:

$$Ce^{3+}(\%) = \frac{v_0+v_1+u_0+u_1}{\sum_i(v_i+u_i)} \times 100\%$$

Supplementary Table 4 Structural information based on the fitted EXAFS results for Pt_e-300A and Pt_e catalysts using PtO_2 and Pt foil as the reference.

**Samples**	**Shell**	**CN** [a]	**R(Å)** [b]	**ΔE_0 (eV)** [c]	**$\Delta\sigma^2$(Å^2)** [d]	**R factor** [e]
Pt_e	Pt-O	4.2 (±0.2)	2.00 (±0.013)	7.4 (±1.4)	0.003 (±0.001)	0.0046
Pt_e-300A	Pt-O	1.5 (±0.1)	2.00 (±0.015)	6.5 (±0.6)	0.003 (±0.001)	0.0032
	Pt-Pt	3.6 (±0.3)	2.74 (±0.022)	9.5 (±2.3)	0.005 (±0.001)	
	Pt-O-Pt	3.8 (±0.4)	2.95 (±0.130)	10.1 (±2.7)	0.004 (±0.001)	
PtO_2	Pt-O	6	2.02 (±0.006)	4.2 (±0.8)	0.003 (±0.001)	0.0071
	Pt-Pt	6	3.08 (±0.010)	7.0 (±2.4)	0.003 (±0.001)	
Pt foil	Pt-Pt	12	2.76 (±0.003)	2.9 (±0.5)	0.003 (±0.001)	0.0022

[a] Coordination number;

[b] Bond length;

[c] Inner potential correction;

[d] Debye-Waller factor;

[e] Goodness-of-fit index.

Supplementary Table 5 Summarization of band positions and vibration modes of the surface adsorbed species during C_3H_6 oxidation.

**Species**	**Vibration mode**	**Wavenumber (cm^{-1})**	**Ref.**
Gaseous propylene	$\nu(C{=}C)$	1658, 1660	[24,25]
	$\delta(CH_2)$	1265, 1274	
Adsorbed propylene	$\nu(C{=}C)$	1622, 1627	[26]
Acetate	$\nu_s(COO^-)$	1459, 1463	[27,28]
	$\delta_s(CH_3)$	1395, 1405	
	$\nu_a(COO^-)$	1587	
Acrolein	$\delta(C\text{-}H)$	1240, 1268	[29,30]
Acrylate	$\nu(C\text{-}C)$	1284, 1288	[31,32]
	$\nu(C{=}C)$	1640	

Supplementary Table 6 Kinetic parameters collected by multiple linear regressions of the tested consumption rate for C_3H_6 oxidation on Pt_e-300A catalysts at 162 °C.

Samples	K_1 (unitless)	K_2 (unitless)	K_3 (unitless)	k_4 (mmol mol^{-1} s^{-1})	R^2
Pt_e-300A	1.52×10^{-5}	5.74×10^{-1}	8.72×10^{-3}	1.11×10^{3}	0.993

Supplementary Table 7 The change of DFT-calculated free energy for C_3H_6 oxidation over H_2-activated Pt/CeO_2 catalysts.

**Steps**	**Structures in the reaction**	**Relative free energy (eV)**
i	O_2 adsorption	-0.02
TS-1		0.08
ii	O_2* dissociation	-0.45
iii	C_3H_6 adsorption	-1.03
TS-2		0.50
iv	O-facilitated dehydrogenation (C_3H_5*)	-0.35
TS-3		0.34
v	O-facilitated dehydrogenation (C_3H_4*)	-1.64
TS-4		-0.63
vi	C_3H_4* coupled O*	-0.66
TS-5		0.17
vii	Formation of acrolein	-1.98
viii	O_2 adsorption	-2.00
TS-6		-1.90
ix	O_2* dissociation	-2.44
TS-7		-1.73
x	O-facilitated dehydrogenation (C_3H_3O*)	-4.67
TS-8		-3.62
xi	Formation of acrylate	-5.16
Final	Generation of CO_2* and H_2O*	-18.21

Supplementary Table 8 The comparison of the activation energy barrier for Langmuir-Hinshelwood and Mars-van Krevelen models for C_3H_6 oxidation over H_2-activated Pt/CeO_2 catalysts.

Steps	Activation energy barrier (eV)	
	**Langmuir-Hinshelwood**	**Mars-van Krevelen**
$O_2* + * \rightarrow 2O*$	0.10	0.22
$O* + O_v \rightarrow O_L + *$	-	0.36
$C_3H_6* + O* \rightarrow C_3H_5* + OH*$	1.53	1.68
$OH* + OH* \rightarrow H_2O* + O*$	1.01	0.37

Supplementary Table 9 Parameters used in the analysis for Mears and Weisz-Prater criteria.

Items	Value	Unit	Description
d_t	5×10^{-3}	m	Diameter of reactor tube
L_b	5×10^{-3}	m	Length of catalyst bed
d_p	4.5×10^{-8}	m	Diameter of catalyst particle
ΔHr	-1.926×10^{6}	J/mol	Reaction heat of C_3H_6
R_g	8.314	J mol^{-1} K^{-1}	Ideal gas constant
R_p	2.25×10^{-8}	m	Radius of catalyst particles
E_a	1.115×10^{5}	J mol^{-1}	Apparent activation energy of C_3H_6 oxidation
λ	0.0302	W m^{-1} K^{-1}	Thermal conductivity of the reactant gases: $\lambda=0.001\lambda_{C3H6}+0.1\lambda_{O2}+0.899\lambda_{N2}$
λ_{C3H6}	0.015	W m^{-1} K^{-1}	Thermal conductivity of the gaseous C_3H_6
λ_{O2}	0.032	W m^{-1} K^{-1}	Thermal conductivity of the gaseous O_2
λ_{N2}	0.030	W m^{-1} K^{-1}	Thermal conductivity of the gaseous N_2
h	1.342×10^{6}	W m^{-2} K^{-1}	Gas-solid heat transfer coefficient: $h=2\lambda/d_p$
T_b	435	K	Bulk phase temperature of C_3H_6 flow
r_{C3H6}	5736.5	mol m^{-3} s^{-1}	Consumption rate of C_3H_6 per catalyst volume
$C_{C3H6,b}$	0.045	mol m^{-3}	C_3H_6 concentration in the bulk gas phase
$C_{C3H6,s}$	0.0225	mol m^{-3}	C_3H_6 concentration at the surface of catalyst
$D_{C3H6\text{-}N2}$	2.04×10^{-5}	m^{2} s^{-1}	Gaseous diffusion coefficient of C_3H_6 into N_2
D_E	2.18×10^{-6}	m^{2} s^{-1}	Effective gaseous diffusion coefficient
k_c	906.7	m s^{-1}	Mass transfer coefficient of the reactant gases: $k_c=2D_{C3H6\text{-}N2}/d_p$

Supplementary Note 13:

The mass and heat limitations were estimated *via* Mears and Weisz-Prater analysis with the selection of 50% C_3H_6 conversion at 162 °C.

Initially, the plug-flow behavior of the flowing reactant gases was confirmed:

$$\frac{L_b}{d_p} = \frac{5\times10^{-3}}{4.5\times10^{-8}} = 1.11\times10^{5} \gg 50$$

$$\frac{d_t}{d_p} = \frac{5\times10^{-3}}{4.5\times10^{-8}} = 1.11\times10^{5} \gg 10$$

Mears criterion for intraparticle and interphase heat transfer:

The intraparticle heat transfer could be neglected if $\frac{|\Delta H_R| r_{C3H6} R_p^2 E_a}{R_g \lambda T_s^2} < 0.15$:

$$\frac{|\Delta H_R| r_{C3H6} R_p^2 E_a}{R_g \lambda T_s^2} = \frac{1.926\times10^{6}\times5736.5\times(2.25\times10^{-8})^2\times1.115\times10^{5}}{8.314\times0.0302\times(435)^2} = 1.31\times10^{-5} \ll 0.15$$

The absence of interphase (external) heat transfer limitation was ensured if $\frac{|\Delta H_R| r_{C3H6} R_p E_a}{R_g h T_b^2} < 0.15$:

$$\frac{|\Delta H_R| r_{C3H6} R_p E_a}{R_g h T_b^2} = \frac{1.926\times10^{6}\times5736.5\times2.25\times10^{-8}\times1.115\times10^{5}}{8.314\times1.342\times10^{6}\times(435)^2} = 1.31\times10^{-5} \ll 0.15$$

The external mass transport limitation could be ignored if $\frac{r_{C3H6} R_p}{k_c C_{C3H6,b}} < 0.15$:

$$\frac{r_{C3H6} R_p}{k_c C_{C3H6,b}} = \frac{5736.5\times2.25\times10^{-8}}{906.7\times0.045} = 3.16\times10^{-6} \ll 0.15$$

The internal mass transport limitation was absent if $C_{WP} = \frac{r_{C3H6} R_p^2}{D_E C_{C3H6,s}} < 1$ based on the Weisz-Prater criterion:

$$C_{WP} = \frac{r_{C3H6} R_p^2}{D_E C_{C3H6,s}} = \frac{5736.5\times(2.25\times10^{-8})^2}{2.18\times10^{-6}\times0.0225} = 5.92\times10^{-5} \ll 1$$

According to the above-calculated results, the heat and mass transport limitations could be excluded in the experimental condition of C_3H_6 oxidation over the Pt_e-300A catalysts.

Supplementary References

1 Nie, L. *et al.* Activation of surface lattice oxygen in single-atom Pt/CeO_2 for low-temperature CO oxidation. *Science* **358**, 1419-1423, (2017).

2 Thommes, M. *et al.* Physisorption of gases, with special reference to the evaluation of surface area and pore size distribution (IUPAC Technical Report). *Pure Appl. Chem.* **87**, 1051-1069, (2015).

3 Romeo, M., Bak, K., El Fallah, J., Le Normand, F. & Hilaire, L. XPS Study of the reduction of cerium dioxide. *Surf. Interface Anal.* **20**, 508-512, (1993).

4 Pfau, A. & Schierbaum, K. D. The electronic structure of stoichiometric and reduced CeO_2 surfaces: an XPS, UPS and HREELS study. *Surf. Sci.* **321**, 71-80, (1994).

5 Boaro, M., Vicario, M., de Leitenburg, C., Dolcetti, G. & Trovarelli, A. The use of temperature-programmed and dynamic/transient methods in catalysis: characterization of ceria-based, model three-way catalysts. *Catal. Today* **77**, 407-417, (2003).

6 Ke, J. *et al.* Strong local coordination structure effects on subnanometer PtO_x clusters over CeO_2 nanowires probed by low-temperature CO oxidation. *ACS Catal.* **5**, 5164-5173, (2015).

7 Ding, K. *et al.* Identification of active sites in CO oxidation and water-gas shift over supported Pt catalysts. *Science* **350**, 189-192, (2015).

8 Hao, H. *et al.* Robust Pt@TiO_x/TiO_2 catalysts for hydrocarbon combustion: effects of Pt-TiO_x interaction and sulfates. *ACS Catal.* **10**, 13543-13548, (2020).

9 Lin, J. *et al.* Elucidating water's place in catalytic C_3H_6 combustion over Pt@TiO_x/TiO_2 with super-hydrophilic silica-modified surface. *Appl. Catal. B: Environ.* **324**, 122234, (2023).

10 Fang, Y. *et al.* Oxygen vacancy-governed opposite catalytic performance for C_3H_6 and C_3H_8 combustion: the effect of the Pt electronic structure and chemisorbed oxygen species. *Environ. Sci. Technol.* **56**, 3245-3257, (2022).

11 Wan, J., Ran, R., Li, M., Wu, X. & Weng, D. Effect of acid and base modification on the catalytic activity of Pt/Al_2O_3 for propene oxidation. *J. Mol. Catal. A: Chem.* **383-384**, 194-202, (2014).

12 Yang, A.-C. *et al.* Insights and comparison of structure–property relationships in propane and propene catalytic combustion on Pd- and Pt-based catalysts. *J. Catal.* **401**, 89-101, (2021).

13 Jiang, Y. *et al.* Enhanced catalytic activity in propene oxidation over NaZSM-5 zeolite-supported Pt nanoparticles by increasing the zeolite Si/Al ratio. *Catal. Today* **355**, 476-481, (2020).

14 Liu, C. *et al.* Preparation of platinum nanocatalysts from mixed-valence precursors: investigation on the size effects of complete oxidation of propene. *J. Mater. Chem. A*, (2025).

15 Tan, W. *et al.* Transformation of highly stable Pt single sites on defect engineered ceria into robust Pt clusters for vehicle emission control. *Environ. Sci. Technol.* **55**, 12607-12618, (2021).

16 Jiang, D. *et al.* Tailoring the local environment of platinum in single-atom Pt_1/CeO_2 catalysts for robust low-temperature CO oxidation. *Angew. Chem. Int. Ed.* **60**, 26054-26062, (2021).

17 Tan, W. *et al.* Fine-tuned local coordination environment of Pt single atoms on ceria controls catalytic reactivity. *Nat. Commun.* **13**, 7070, (2022).

18 Liu, X. *et al.* Activation of subnanometric Pt on Cu-modified CeO_2 *via* redox-coupled atomic layer deposition for CO oxidation. *Nat. Commun.* **11**, 4240, (2020).

19 Wang, H. *et al.* Surpassing the single-atom catalytic activity limit through paired Pt-O-Pt ensemble built from isolated Pt_1 atoms. *Nat. Commun.* **10**, 3808, (2019).

20 Xie, S. *et al.* Pt atomic single-layer catalyst embedded in defect-enriched ceria for efficient CO oxidation. *J. Am. Chem. Soc.* **144**, 21255-21266, (2022).

21 Xiong, H. *et al.* Engineering catalyst supports to stabilize PdO_x two-dimensional rafts for water-tolerant methane oxidation. *Nat. Catal.* **4**, 830-839, (2021).

22 Wang, W. *et al.* Insights into different eeaction behaviors of propane and CO oxidation over Pt/CeO_2 and Pt/Nb_2O_5: the crucial roles of support properties. *J. Phys. Chem. C* **125**, 19301-19310, (2021).

23 Pereira-Hernández, X. I. *et al.* Tuning Pt-CeO_2 interactions by high-temperature vapor-phase synthesis for improved reducibility of lattice oxygen. *Nat. Commun.* **10**, 1358, (2019).

24 Lord, R. C. & Venkateswarlu, P. The infrared spectra of propylene and propylene-d6*. *J. Opt. Soc. Am.* **43**, 1079-1085, (1953).

25 Driscoll, D. M. *et al.* Binding sites, geometry, and energetics of propene at nanoparticulate Au/TiO_2. *J. Phys. Chem. C* **121**, 1683-1689, (2017).

26 Zaera, F. & Chrysostomou, D. Propylene on Pt(111). *Surf. Sci.* **457**, 89-108, (2000).

27 Hazlett, M. J., Moses-Debusk, M., Parks, J. E., Allard, L. F. & Epling, W. S. Kinetic and mechanistic study of bimetallic Pt-Pd/Al_2O_3 catalysts for CO and C_3H_6 oxidation. *Appl. Catal. B: Environ.* **202**, 404-417, (2017).

28 Vratny, F., Rao, C. N. R. & Dilling, M. Infrared spectra of metal acetates. *Anal. Chem.* **33**, 1455-1455, (1961).

29 Finocchio, E., Busca, G., Lorenzelli, V. & Willey, R. J. FTIR studies on the selective oxidation and combustion of light hydrocarbns at metal oxide surfaces. Propane and propene oxidation on $MgCr_2O_4$. *J. Chem. Soc., Faraday Trans.* **90**, 3347-3356, (1994).

30 Harris, R. K. Vibrational assignments for glyoxal, acrolein and butadiene. *Spectrochim. Acta* **20**, 1129-1141, (1964).

31 Finocchio, E., Busca, G., Lorenzelli, V. & Escribano, V. S. FTIR studies on the selective oxidation and combustion of light hydrocarbons at metal oxide surfaces. Part 2.-Propane and propene oxidation on Co_3O_4. *J. Chem. Soc., Faraday Trans.* **92**, 1587-1593, (1996).

32 Finocchio, E., J. Willey, R., Busca, G. & Lorenzelli, V. FTIR studies on the selective oxidation and combustion of light hydrocarbons at metal oxide surfaces Part 3.-Comparison of the oxidation of C3 organic compounds over Co_3O_4, $MgCr_2O_4$ and CuO. *J. Chem. Soc., Faraday Trans.* **93**, 175-180, (1997).
