# Supplementary material for: Temperature-driven mechanistic transition in propylene oxidation over Pt/CeO2 ensemble catalysts
Source: Nat Commun. 2025 Oct 16;16:9199. doi: 10.1038/s41467-025-64243-y (PMC12532794; doi:10.1038/s41467-025-64243-y)
Supplement: Supplementary file 4 — Source Data [file 41467_2025_64243_MOESM4_ESM.zip › Source Data/Source Data_Supplementary Information.pptx]

## Slide 1
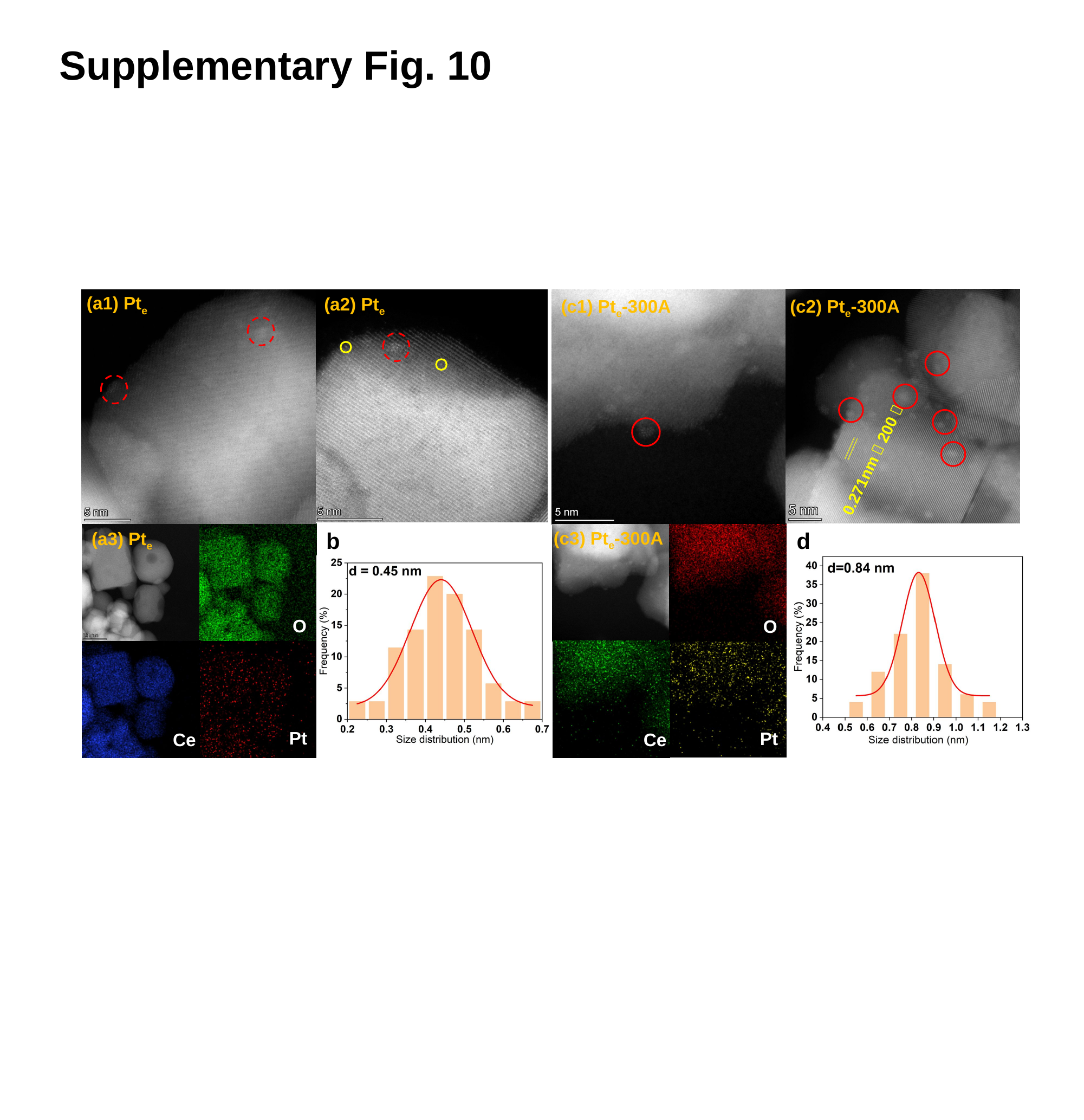

Supplementary Fig. 10
(a1) Pte
(a2) Pte
(c2) Pte-300A
0.271nm（200）
(c1) Pte-300A
b
d
(c3) Pte-300A
O
Pt
Ce
(a3) Pte
O
Pt
Ce

## Slide 2
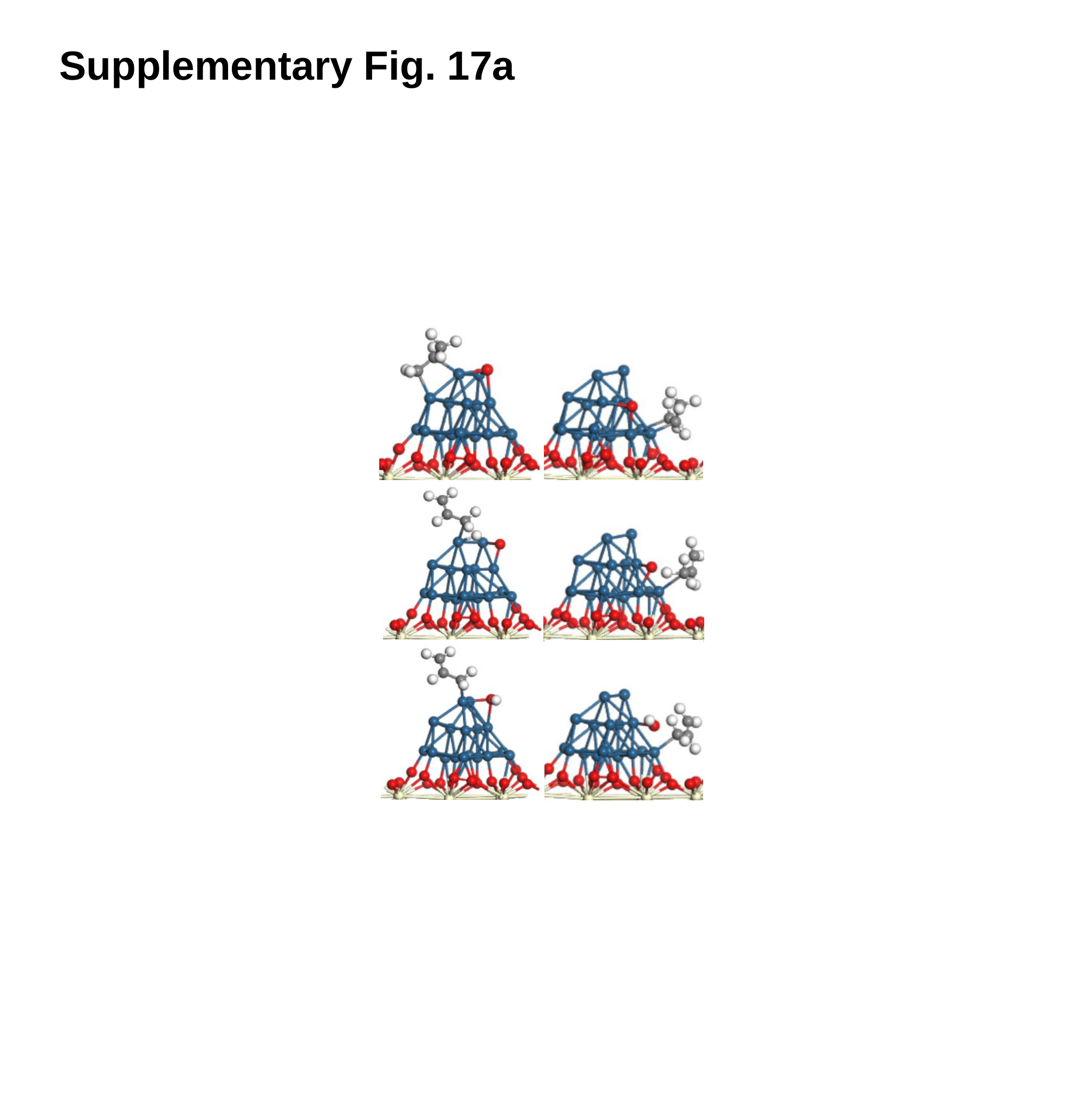

Supplementary Fig. 17a

## Slide 3
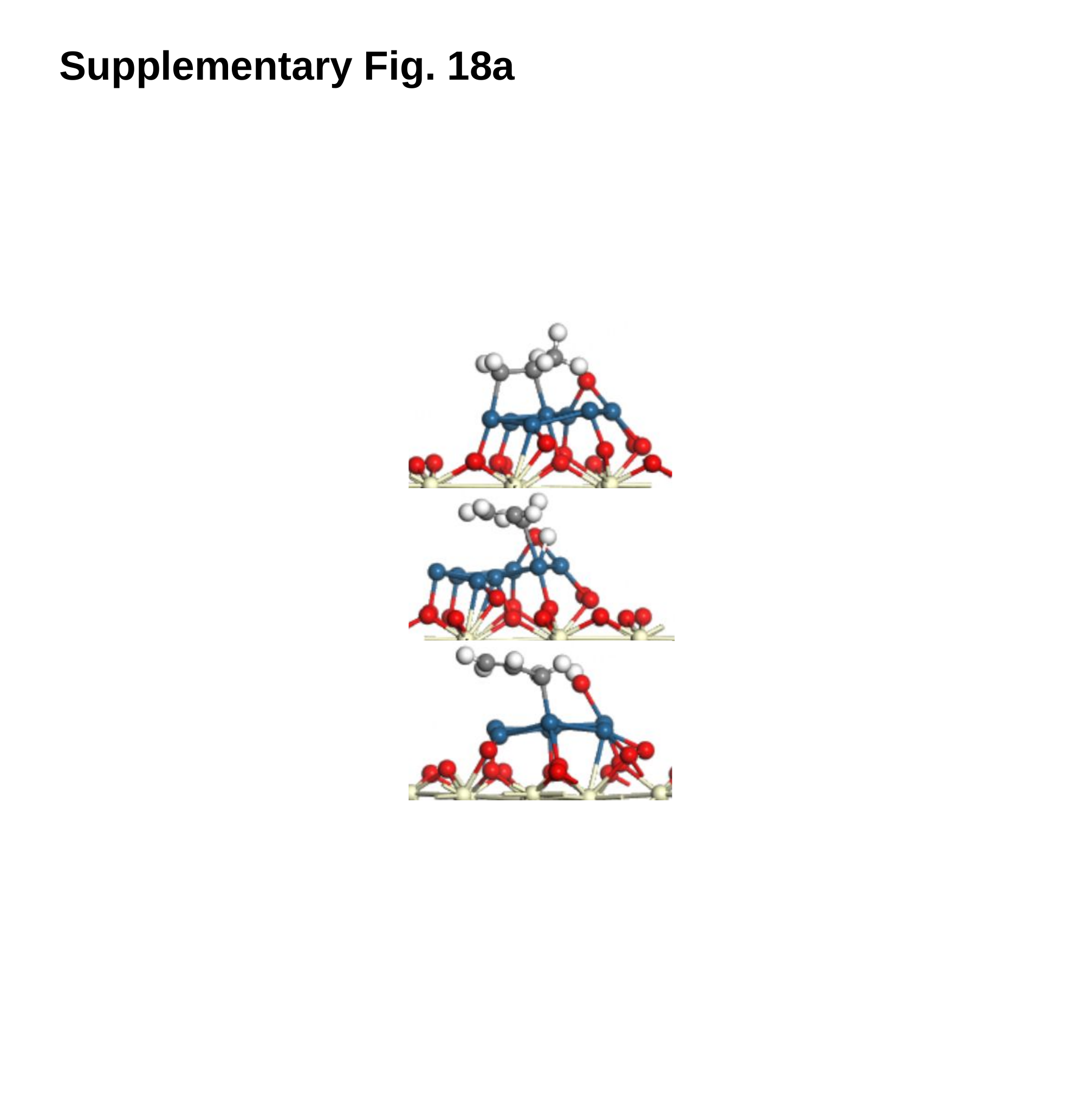

Supplementary Fig. 18a

## Slide 4
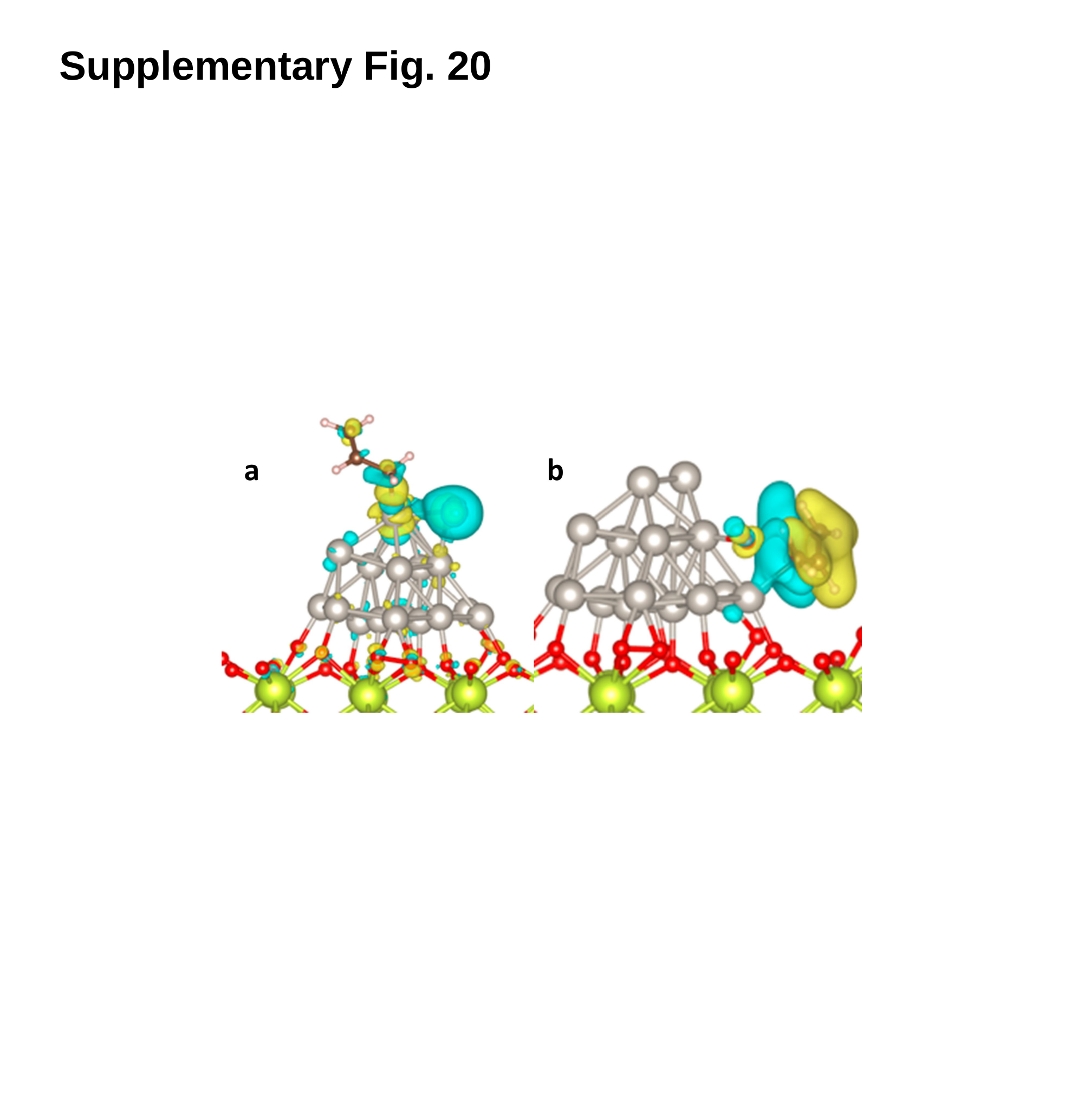

Supplementary Fig. 20

## Slide 5
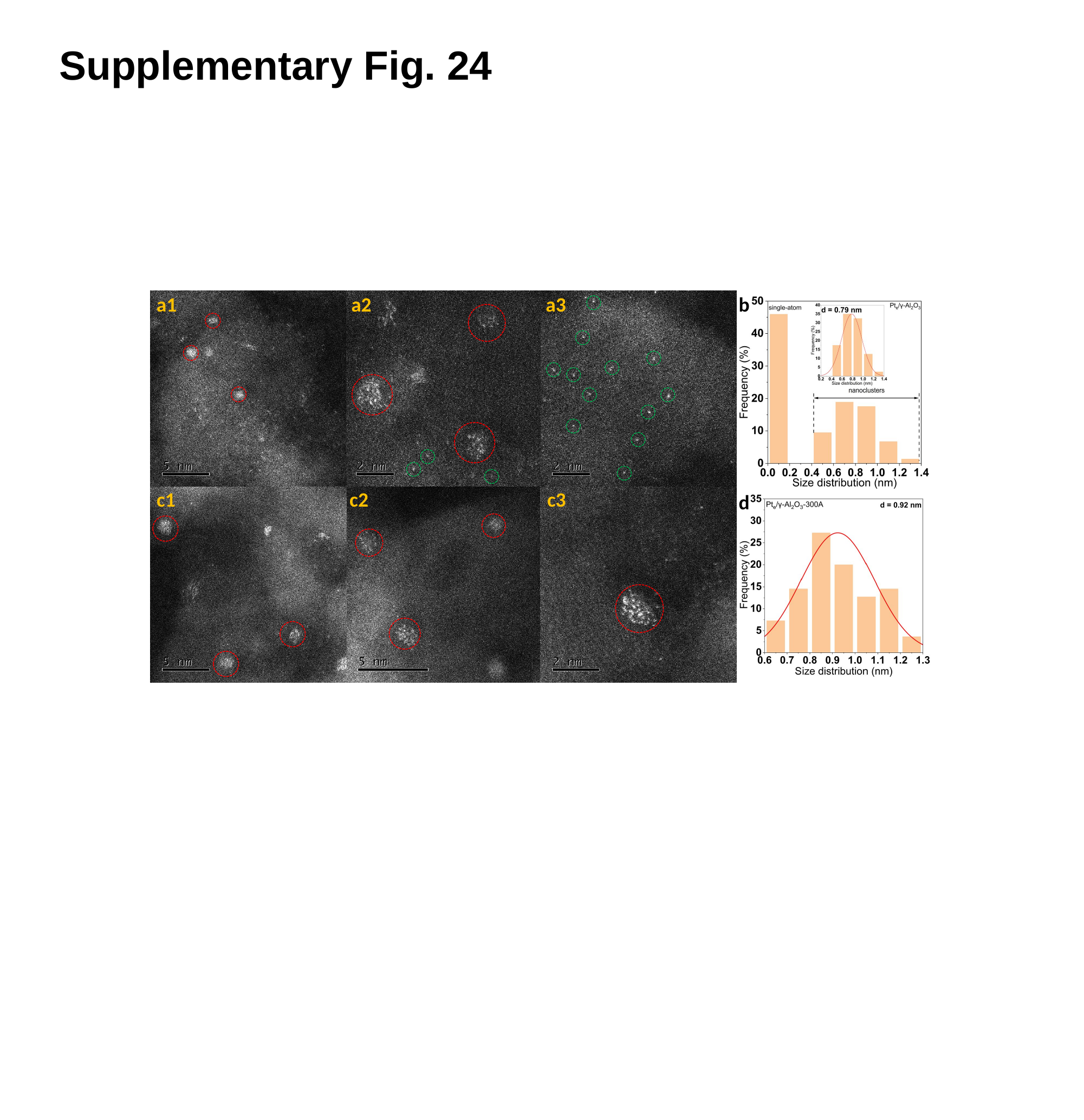

Supplementary Fig. 24
a1
a2
a3
b
c1
c2
c3
d
