# Supplementary figures and images for: Temperature-driven mechanistic transition in propylene oxidation over Pt/CeO2 ensemble catalysts
Source: Nat Commun. 2025 Oct 16;16:9199. doi: 10.1038/s41467-025-64243-y (PMC12532794; doi:10.1038/s41467-025-64243-y)

## Slide 1
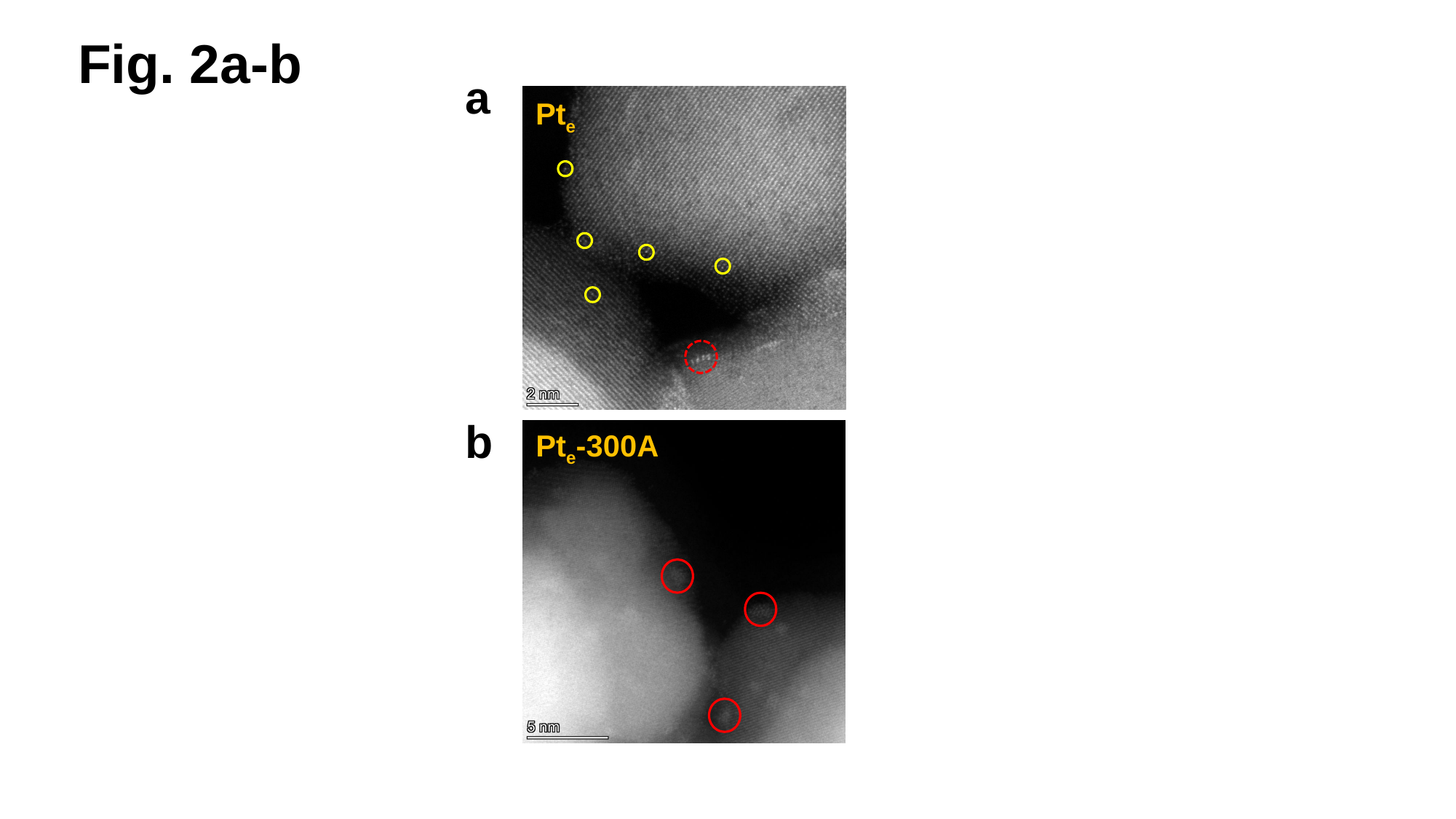

Fig. 2a-b
a
Pte
b
Pte-300A

## Slide 2
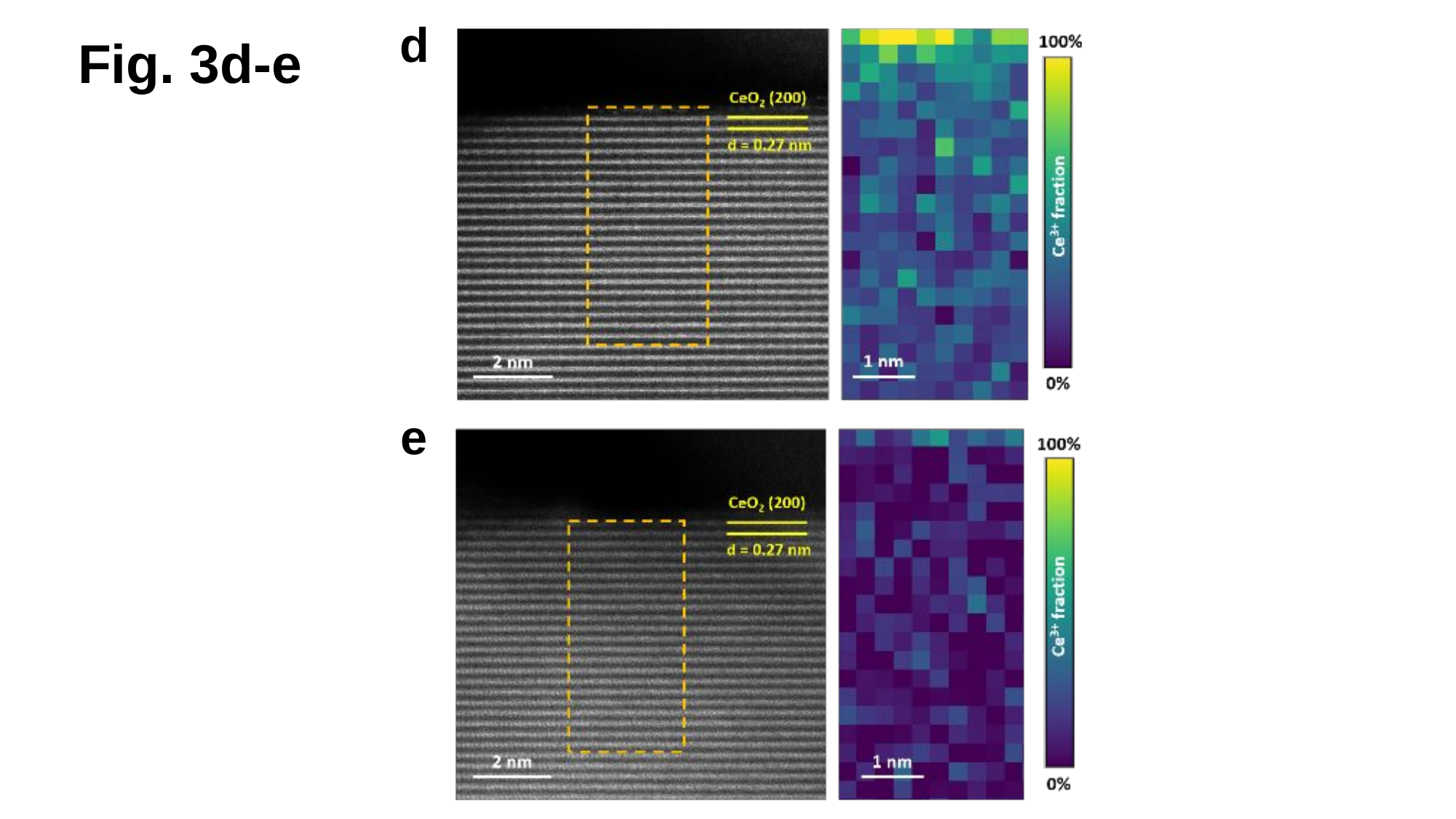

d
Fig. 3d-e
e

## Slide 3
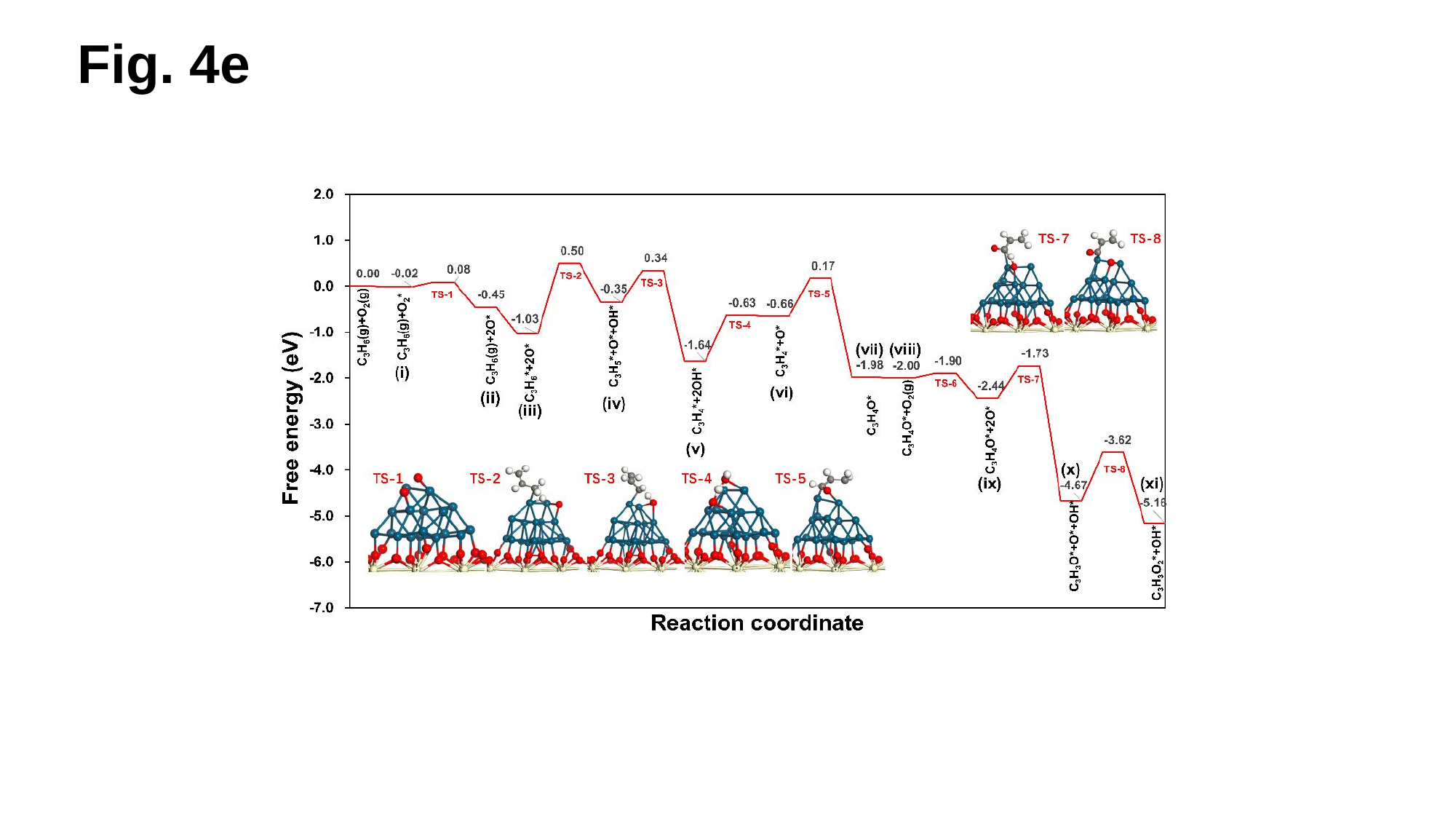

Fig. 4e

## Slide 4
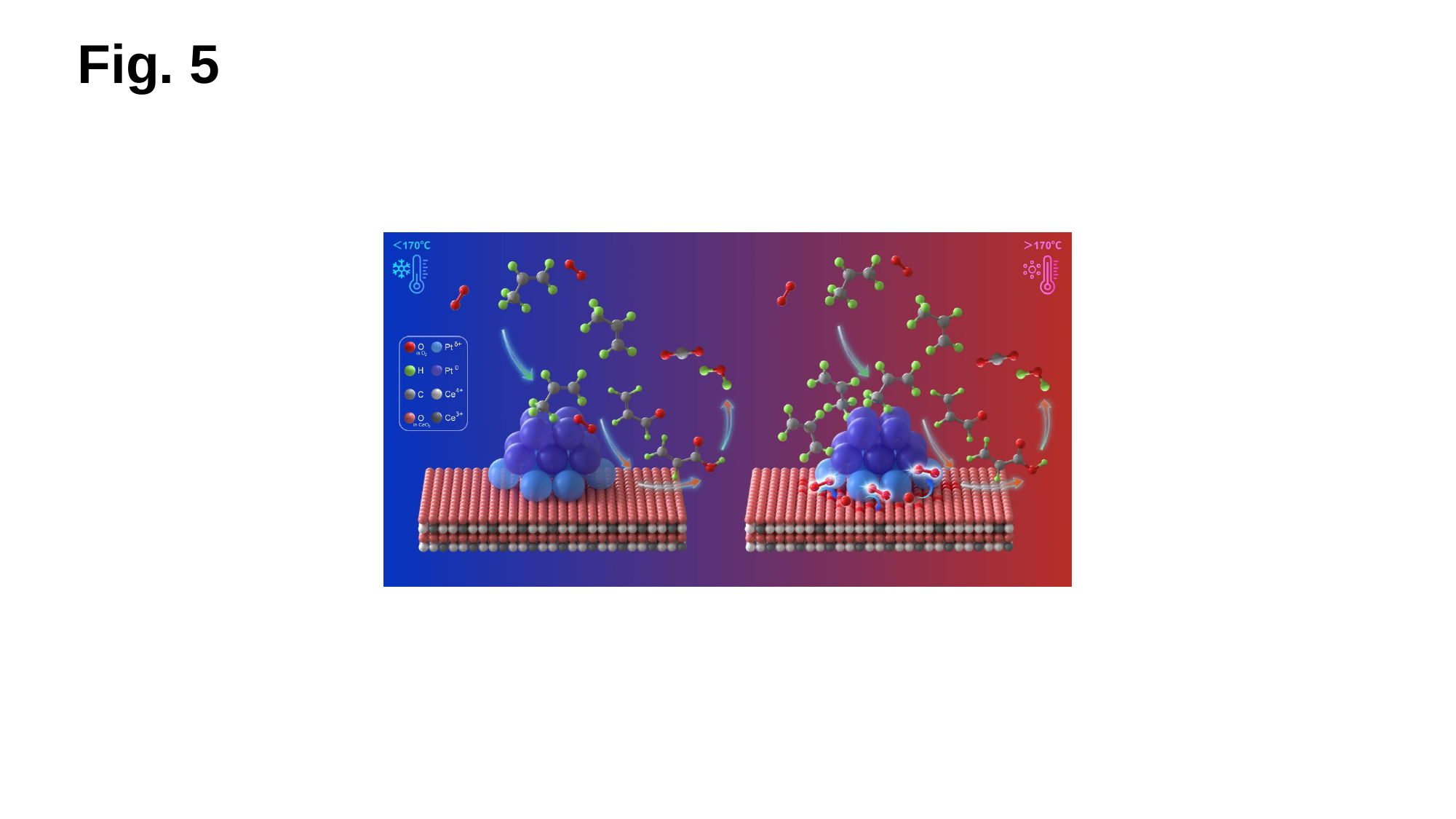

Fig. 5

Supplement: Supplementary file 4 — Source Data [file 41467_2025_64243_MOESM4_ESM.zip › Source Data/Source Data_Main Manuscript.pptx]
